# Synthesis of 4-Arylselanyl-1*H*-1,2,3-triazoles from Selenium-Containing Carbinols

**DOI:** 10.3390/molecules26082224

**Published:** 2021-04-12

**Authors:** Francesca Begini, Renata A. Balaguez, Allya Larroza, Eric F. Lopes, Eder João Lenardão, Claudio Santi, Diego Alves

**Affiliations:** 1 Group of Catalysis, Synthesis and Organic Green Chemistry, Department of Pharmaceutical Sciences University of Perugia Via del Liceo 1, 06123 Perugia, Italy; francescabegini@hotmail.it (F.B.); claudio.santi@unipg.it (C.S.); 2 LASOL-CCQFA, Universidade Federal de Pelotas-UFPel, P.O. Box 354, 96010-900 Pelotas, Brazil; renata.balaguez@gmail.com (R.A.B.); allya.larroza@gmail.com (A.L.); eric.francislopes@gmail.com (E.F.L.); lenardao@ufpel.edu.br (E.J.L.)

**Keywords:** selenium, 1,2,3-triazoles, click chemistry, cycloaddition, carbinols, heterocycles

## Abstract

In this work, we present a simple way to achieve 4-arylselanyl-1*H*-1,2,3-triazoles from selenium-containing carbinols in a one-pot strategy. The selenium-containing carbinols were used as starting materials to produce a range of selanyl-triazoles in moderate to good yields, including a quinoline and Zidovudine derivatives. One-pot protocols are crucial to the current concerns about waste production and solvent consumption, avoiding the isolation and purification steps of the reactive terminal selanylalkynes. We could also isolate an interesting and unprecedented by-product with one alkynylselenium moiety connected to the triazole.

## 1. Introduction

Triazoles are a significant class of heterocycles which have received considerable attention because of their application in materials science, medicinal chemistry and organic synthesis [1,2,3]. Particularly, 1,2,3-triazoles derivatives exhibit a broad spectrum of biological properties, such as anti-inflammatory, antifungal, antibacterial, anticancer, antivirus and antituberculosis [4,5,6,7,8,9,10,11,12,13]. 1,2,3-Triazoles derivatives are an important connecting group, linking a broad range of substituted substrates in a simple fashion, being used as peptide mimetics [14,15]. Inspired in the Huisgen [3 + 2] cycloaddition reaction of an organic azide and a terminal alkyne [16], a number of catalytic strategies employing transition metals have been used to address the reactivity and selectivity issues inherent to the seminal strategy [17,18,19,20,21,22,23,24,25,26]. In addition, recent studies have been directed toward the development of metal-free methodologies for triazole synthesis. Organocatalytic approaches involving [3 + 2] cycloaddition have been reported for the synthesis of functionalized 1,2,3-triazoles [27,28,29,30,31,32,33,34].

Despite the significant advances toward the synthesis of highly substituted 1,2,3-triazoles, the need of a deep study on the combinations of substrates for the synthesis of highly functionalized and complex structures is still an open issue. In this sense, organoselanyl-triazoles constitute an interesting class of molecules, which combine the importance of a triazole nucleus [1,2,3] with an organoselenium moiety [35,36,37,38]. Selenium is an essential nutrient for mammals, playing important roles in metabolic pathways [39,40], and the interest in selenium pharmacology [41,42,43,44,45,46] and chemistry [47,48,49] has increased in this century.

**Figure 1:**
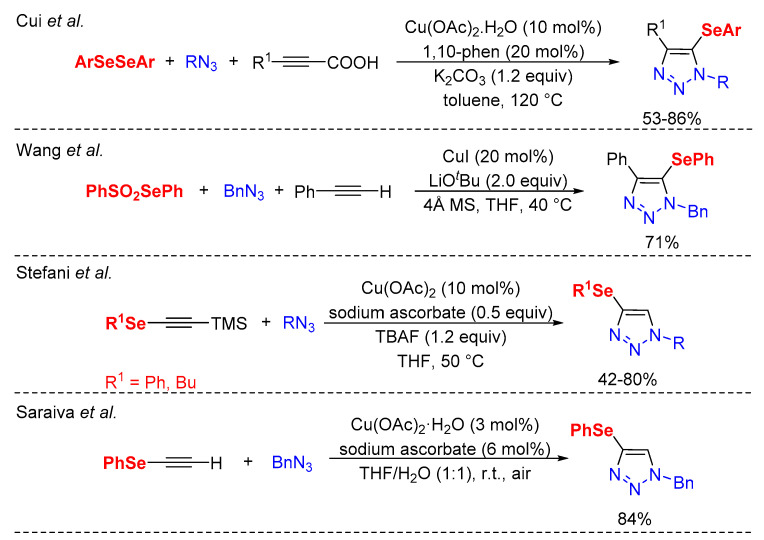
Previous protocols to prepare 5-arylselanyl- and 4-arylselanyl-1*H*-1,2,3-triazoles.

Several methodologies have been reported for the selective synthesis of a range of 1,2,3-triazole scaffolds containing an organoselenium moiety [50,51,52]. However, only a few procedures to directly prepare 5-arylselanyl- and 4-arylselanyl-1*H*-1,2,3-triazoles have been described (Figure 1). For example, Cui et al. developed a simple and efficient method for the preparation of 5-arylselanyl-1*H*-1,2,3-triazoles from propiolic acids, diselenides and azides, in which a selanylalkyne was firstly formed via decarboxylative reactions, followed by the intermolecular copper-catalyzed azide-alkyne cycloaddition reaction (CuAAC) to afford the desired products [53]. Wang et al. described the use of PhSO_2_SePh as an electrophile in the copper (I)-catalyzed interrupted click reaction of phenylacetylene with benzylazide, giving 5-arylselanyl-1*H*-1,2,3-triazole in 71% yield [54]. Manarin et al. developed a general method for the synthesis of 4-arylselanyl-1*H*-1,2,3-triazoles via a CuAAC reaction between organic azides and a terminal selanylalkyne, generated by the in situ deprotection of the silyl group [55]. The synthesis of 1-benzyl-4-(phenylselanyl)-1*H*-1,2,3-triazole was described by Saraiva et al., in which ethynyl(phenyl)selenide underwent CuAAC with benzylazide to give the product in 84% yield [56]. However, for the synthesis of ethynyl(phenyl)selenide, the protocol available at the time to achieve such starting material was described by Braga et al., dating from 1994 [57]. Recently, we have developed an alternative way to prepare these terminal alkynes containing selenium and sulfur, starting from chalcogen-containing alkynyl carbinols [58]. In this study, during the preparation of the terminal selanylalkynes, it was observed that in air without solvent, these compounds showed signals of decomposition. Furthermore, we observed that in a hexane solution, the terminal selanylalkynes were stable in the presence of air. With these observations in mind, we wondered if selanylalkynylcarbinols could serve as starting materials for the synthesis of a range of 4-arylselanyl-1*H*-1,2,3-triazoles in a one-pot procedure.

**Scheme 1:**
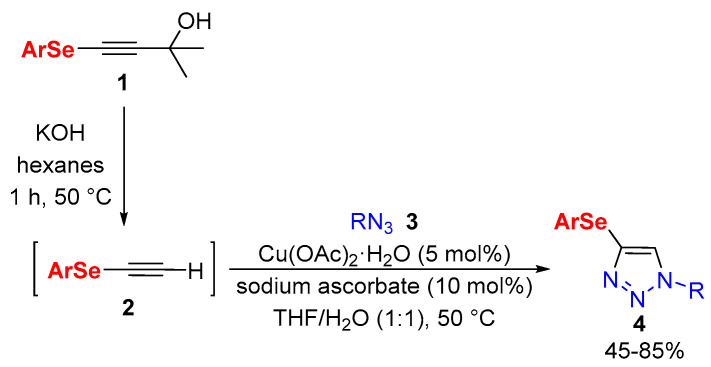
Synthesis of 4-arylselanyl-1*H*-1,2,3-triazoles from selenium-containing carbinols.

In view of the above, and in continuation to our research endeavors in the development of efficient and selective methods to access functionalized selanyl-1,2,3-triazoles, we report herein a one-pot strategy to prepare 4-arylselanyl-1*H*-1,2,3-triazoles, starting from easily available and bench-stable selanylalkynylcarbinols and organic azides (Scheme 1).

## 2. Results and Discussion

Initial experiments to optimize the reaction conditions were carried out using 2-methyl-4-(phenylselanyl)but-3-yn-2-ol **1a** and 1-azido-4-chlorobenzene **3a** as standard reaction substrates (Table 1). The key step of the protocol involved the deprotection of the hydroxypropargyl selenide **1a** (1 mmol) to give the terminal selanylalkyne intermediate **2a** according to a retro-Favorskii reaction mechanism. For this reaction, we used our previously optimized conditions (KOH/hexanes, 50 °C) [58], and after 1 h, the propargyl alcohol **1a** (monitored by TLC) was completely consumed. Then, the crude reaction mixture was allowed to reach room temperature, and a 1:1 mixture of THF/H_2_O (1.0 mL) was added, followed by 1-azido-4-chlorobenzene **3a** (0.5 mmol), sodium ascorbate (10 mol%) and Cu(OAc)_2_·H_2_O (5 mol%). The resulting mixture was then stirred at 50 °C until all the azide **3a** was not observable anymore by TLC, 8.0 h. Under these conditions, the expected 4-phenylselanyl-1*H*-1,2,3-triazole **4a** was obtained in 85% yield.

From this promising result, some additional experiments were conducted, aiming to increase the yield of **4a** while reducing the reaction time (Table 1). Firstly, different copper species (CuI, CuO_nps_ and CuCl_2_) were tested under the same conditions, but in all the cases we observed lower yields than that obtained using Cu(OAc)_2_·H_2_O (Table 1, entry 1 vs. entries 2–5). For instance, the use of CuI gave **4a** in 60% yield under the conditions of entry 1, and only 32% using Et_3_N in the place of sodium ascorbate and in DMSO as the solvent (entries 2 and 3). Only traces of **4a** were observed using CuO_nps_, while CuCl_2_ afforded the expected product in 75% yield (Table 1, entries 4 and 5). The presence of water in the reaction medium was essential for the success of the reaction once no product was observed using dry THF (Table 1, entry 6). The use of lower amounts of both, sodium ascorbate (6 mol%) and Cu(OAc)_2_·H_2_O (3 mol%), or an argon atmosphere, negatively influenced the reaction, affording **4a** in 40% and 65% yield, respectively (Table 1, entries 7 and 8). The influence of the temperature and the stoichiometry of the reagents was evaluated. At room temperature, the pre-formed terminal selanylacetylene **1a** reacted with azide **3a** to give **4a** in 40% yield (Table 1, entry 9). A moderate result was also observed when equivalent amounts of **2a** and **3a** were reacted, affording **4a** in 50% yield (Table 1, entry 10).

After analyzing these results, we determined that the best reaction conditions were those reported in Table 1, entry 1: after stirring a mixture of the propargyl alcohol **1a** and KOH in hexanes, the resulting in situ formed terminal alkyne **2a** mixed with the azide **3a** (0.5 equiv.) were stirred in the presence of sodium ascorbate (10 mol%) and Cu(OAc)_2_·H_2_O (5 mol%) in a 1:1 mixture of THF and H_2_O as the solvent.

**Scheme 2:**
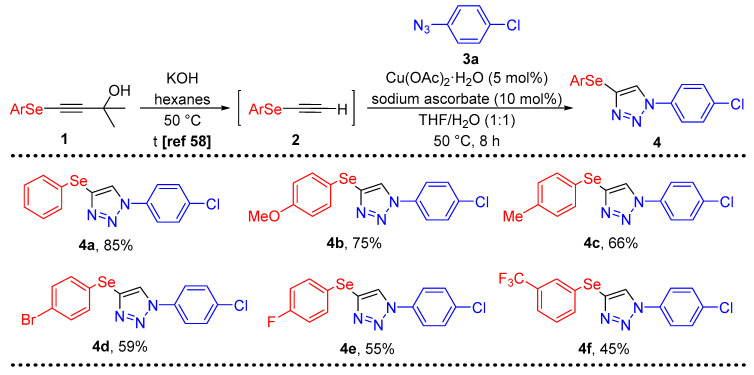
4-Arylselanyl-1*H*-1,2,3-triazoles **4a**-**f**: scope of arylselanyl carbinols **1**.

The scope of the proposed methodology was then extended to differently substituted alkynyl selenides **1b–f**, in the reaction with 1-azido-4-chlorobenzene **3a**, aiming to investigate the generality and limitations of the method (Scheme 2). Interesting, there is a little influence of the electronic effect in the reaction, and the presence of electron-donating groups in the *para*-position of the pendant phenyl increase the reactivity. For instance, electron-rich 4-((4-methoxyphenyl)selanyl)- **1b** (Ar = 4-MeOC_6_H_4_) and 2-methyl-4-(*p*-tolylselanyl)but-3-yn-2-ol **1c** (Ar = 4-MeC_6_H_4_) afforded the respective 4-arylselanyl-1*H*-1,2,3-triazoles **4b** and **4c** in 75% and 66% yield, while the electron-poor one 2-methyl-4-(4-fluoroselanyl)but-3-yn-2-ol **1e** (Ar = 4-FC_6_H_4_) afforded the triazole **4e** in 55% yield. A remarkable result was obtained in the reaction of 2-methyl-4-(4-bromoselanyl)but-3-yn-2-ol **1d** (Ar = 4-BrC_6_H_4_), which afforded the bromo-functionalized triazole **4d** (59% yield), which can be subject to further transformation via Sonogashira cross-coupling reaction. A decrease in yield was observed, however, when the strong electron withdrawing CF_3_ group was present in the *meta*-position. Thus, 2-methyl-4-((3-(trifluoromethyl)phenyl)selanyl)but-3-yn-2-ol **1e** reacted with **3a** under the optimal conditions to afford the expected triazole **4f** in 45% yield (Scheme 2).

**Scheme 3:**
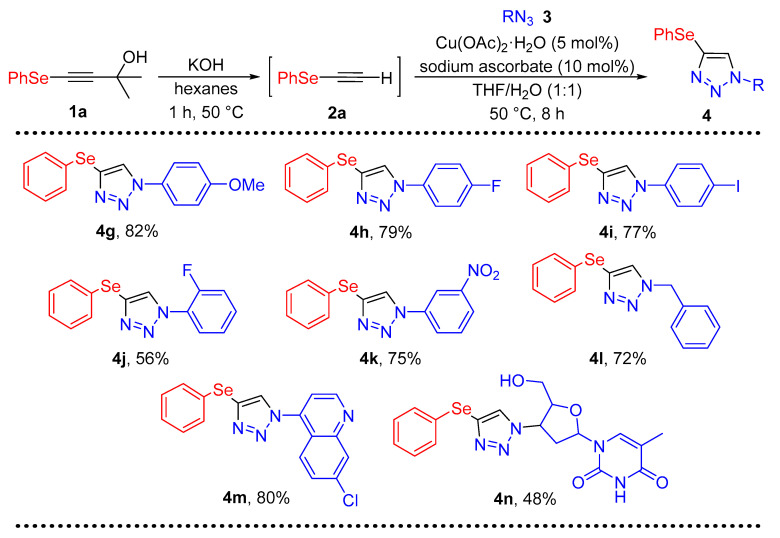
4-Arylselanyl-1*H*-1,2,3-triazoles **4g**-**n**: scope of azides **3**.

Subsequently, we investigated the reactivity of a variety of organic azides **3** with 2-methyl-4-(phenylselanyl)but-3-yn-2-ol **1a** under the best reaction conditions (Scheme 3). As for the alkynyl selenide counterpart, electronic effect does not seem to influence the reactivity of the *para*-substituted aryl azides **3**. For instance, the electron-rich 1-azido-4-methoxybenzene **3b** (R = 4-MeOC_6_H_4_) and the electron-deficient 1-azido-4-fluorobenzene **3c** (R = 4-FC_6_H_4_) afforded the respective triazoles **4g** and **4h** in 82% and 79% yield after reaction with **2a**. A similarly good result was observed from the 4-iodo-substituted azide **3d**, affording the iodo-containing triazole **4i** in 77% yield, which could be subject to further modifications, as mentioned for **4d**. The presence of a fluoro atom at the *ortho*-position, like in **3e** (R = 2-FC_6_H_4_), slightly affected the reactivity, and the respective product **4j** was isolated in 56% yield. Interestingly, the strong electron-withdrawing nitro group positively affected the reaction, and 1-azido-3-nitrobenzene **3f** (R = 3-NO_2_C_6_H_4_) gave **4k** in 75% yield. (Azidomethyl)benzene **3g** was a suitable substrate in the reaction with **2a** (generated in situ from **1a**), affording 1-benzyl-4-(phenylselanyl)-1-1,2,3-triazole **4l** in 72% yield. Molecular hybridization is a valuable strategy in medicinal chemistry, allowing access to potent multitarget drugs [59,60]. In view of the recognized bioactivity of both, organoselenium and triazole units, we decided to explore the functionalization of two known nuclei, 7-chloroquinoline and Zidovudine, which could present interesting pharmacological properties to be explored. Thus, 4-azido-7-chloroquinoline **3h** reacted with **2a** to give the 7-chloroquinoline-derivative **4m** in 80% yield, while the azido-derivative of Zidovudine **3i** was converted to the respective triazole **4n** in 48% yield.

**Scheme 4:**
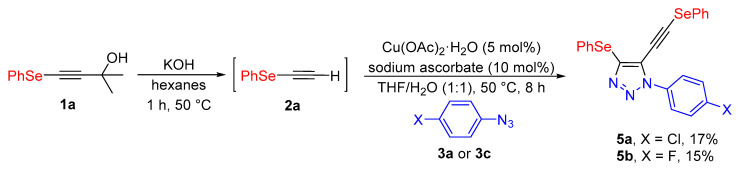
By-products **5a** and **5b** from the reaction of **3a** or **3c** with **2a** generated in situ.

While performing these CuAAc reactions, the formation of a by-product was observed, with a retention factor (RF) in thin layer chromatography remarkably similar to product **4**. This by-product was isolated and characterized as the triazole derived from the reaction of the organyl azide **3** with two equiv. of alkynyl selenide **2a**. Unfortunately, the purification of this by-product is extremely difficult because of the similarity of RF with the main product **4**. Fortunately, the by-products **5a** and **5b** could be isolated, even if in low yields, and were fully characterized (Scheme 4). A possible explanation for the formation of alkynes **5** is the presence of the remaining strong base (KOH), used in the first step of the reaction (the retro-Favorskii of propargyl alcohol **2a**), according to the previously observed by Li, Zhang et al. [61].

## 3. Materials and Methods

Reactions were carried out in a two-necked round-bottomed flask with a Teflon-coated magnetic stirring bar. Solvents and reagents were used as received unless otherwise noted. The reactions were monitored by TLC performed by using Merck silica gel (60 F254), 0.25 mm thickness. For visualization, TLC plates were either placed under UV light, or stained with iodine vapor or 5% vanillin in 10% H_2_SO_4_ under heating. Column chromatography was performed by using Merck silica gel (230–400 mesh). Carbon-13 nuclear magnetic resonance spectra (^13^C NMR) were obtained at 75 MHz on a Bruker DPX 300 spectrometer and at 100 MHz on a Bruker Avance III HD 400 spectrometer. Spectra were recorded in CDCl_3_ solutions. Chemical shifts are reported in ppm, referenced to tetramethylsilane (TMS) as the external reference (^1^H NMR) or to the solvent peak of CDCl_3_ (^13^C NMR). Coupling constants (*J*) are reported in Hertz. Abbreviations to denote the multiplicity of a particular signal are s (singlet), d (doublet), t (triplet), dd (double doublet), q (quartet) and m (multiplet). High resolution mass spectra (HRMS) were recorded on a Bruker Micro TOF-QII spectrometer 10416. Reagents 2-methyl-3-butyn-2-ol and selenium powder were purchased from Sigma-Aldrich. The starting materials selanylalkynylcarbinols were synthesized according to previous literature [58]. ^1^H and ^13^C NMR spectra of all compounds are available in Supplementary Materials.

### General Procedure for the Synthesis of 4-Arylselanyl-1H-1,2,3-triazoles ***4***

Arylselanyl carbinol **1** (1.0 mmol), KOH (1.1 mmol, 0.062 g) and hexanes (3.0 mL) were added to a 25 mL two-necked round-bottomed flask equipped with a reflux condenser. The system was then immersed in a preheated oil bath at 50 °C and stirred at this temperature for 1 to 5 h (the consumption of carbinol **1** was followed by TLC) [58]. Then, 0.5 mmol of the appropriate azide 3, Cu(OAc)_2_·H_2_O (0.025 mmol), sodium ascorbate (0.5 mmol), THF (0.5 mL) and H_2_O (0.5 mL) were added to the reaction flask. The resulting solution was stirred at 50 °C for 8 h. Then, a saturated solution of NH_4_Cl (10 mL) was added, followed by the addition of EtOAc (10 mL). The organic layer was separated, and the aqueous phase was extracted with EtOAc (3× 10 mL), dried over MgSO_4_, and the solvent was evaporated under reduced pressure. The crude product was purified by column chromatography on silica gel with a mixture of hexane/ethyl acetate (9:1) as eluent. Spectral data for the prepared products are listed below.

*1-(4-Chlorophenyl)-4-(phenylselanyl)-1H-1,2,3-triazole* (**4a**): Pale yellow solid, mp: 105–107 °C. Yield: 0.142 g (85%). ^1^H NMR (300 MHz, CDCl_3_) δ: 8.05 (s, 1H), 7.67 (d, *J* = 8.9 Hz, 2H), 7.54–7.47 (m, 4H), 7.26–7.24 (m, 3H). ^13^C NMR (75 MHz, CDCl_3_) δ: 135.0, 134.8, 133.7, 131.9, 129.9, 129.4, 127.6, 126.3, 124.4, 121.6. HRMS Calcd. for C_14_H_10_ClN_3_Se [M + H]^+^: 335.9799. Found: 335.9802.

*1-(4-Chlorophenyl)-4-((4-methoxyphenyl)selanyl)-1H-1,2,3-triazole* (**4b**): Yellow solid, mp: 86–88 °C. Yield: 0.137 g (75%). ^1^H NMR (400 MHz, CDCl_3_) δ: 7.92 (s, 1H), 7.64 (d, *J* = 8.9 Hz, 2H), 7.57 (d, *J* = 8.8 Hz, 2H), 7.47 (d, *J* = 8.9 Hz, 1H), 6.82 (d, *J* = 8.8 Hz, 2H), 3.78 (s, 3H). ^13^C NMR (100 MHz, CDCl_3_) δ: 159.8, 135.4, 135.2, 134.7, 129.9, 125.1, 124.3, 121.6, 119.3, 115.1, 55.3. HRMS Calcd. for C_15_H_12_ClN_3_OSe [M-N_2_ + H]^+^: 337.9843. Found: 337.9843.

*1-(4-Chlorophenyl)-4-(p-tolylselanyl)-1H-1,2,3-triazole* (**4c**): Yellow solid, mp: 74–75 °C. Yield: 0.115 g (66%). ^1^H NMR (400 MHz, CDCl_3_) δ: 7.87 (s, 1H), 7.58 (d, *J* = 8.9 Hz, 2H), 7.42–7.39 (m, 4H), 7.01 (d, *J* = 7.9 Hz, 2H). ^13^C NMR (100 MHz, CDCl_3_) δ: 137.9, 133.7, 132.8, 130.8, 130.2, 130.0, 129.5, 125.6, 124.4, 121.6, 21.0. HRMS Calcd. for C_15_H_12_ClN_3_Se [M-N_2_ + H]^+^: 321.9894. Found: 321.9875.

*4-((4-Bromophenyl)selanyl)-1-(4-chlorophenyl)-1H-1,2,3-triazole* (**4d**): Yellow solid, mp: 46–48 °C. Yield: 0.122 g (59%). ^1^H NMR (400 MHz, CDCl_3_) δ: 7.99 (s, 1H), 7.61 (d, *J* = 8.9 Hz, 2H), 7.43 (d, *J* = 8.9 Hz, 2H), 7.33–7.28 (m, 4H). ^13^C NMR (100 MHz, CDCl_3_) δ: 135.0, 133.5, 132.4, 132.0, 130.0, 129.4, 128.9, 127.6, 126.4, 121.7. HRMS Calcd. for C_14_H_9_BrClN_3_Se [M-N_2_ + H]^+^: 385.8840. Found: 385.8838.

*1-(4-Chlorophenyl)-4-((4-fluorophenyl)selanyl)-1H-1,2,3-triazole* (**4e**): Yellow solid, mp: 48–50 °C. Yield: 0.097 g (55%). ^1^H NMR (400 MHz, CDCl_3_) δ: 8.02 (s, 1H), 7.67 (d, *J* = 8.7 Hz, 2H), 7.56 (dd, *J* = 8.6 and 5.3 Hz, 2H), 7.49 (d, *J* = 8.7 Hz, 2H), 6.97 (t, *J* = 8.6 Hz, 2H). ^13^C NMR (100 MHz, CDCl_3_) δ: 162.6 (d, *J*_C-F_ = 248.1 Hz), 135.0, 134.9, 134.7 (d, *J*_C-F_ = 8.0 Hz), 131.1, 130.0, 125.9, 124.1 (d, *J*_C-F_ = 3.5 Hz), 121.6, 116.6 (d, *J*_C-F_ = 21.6 Hz). HRMS Calcd. for C_14_H_9_ClFN_3_Se [M-N_2_ + H]^+^: 325.9643. Found: 325.9636.

*1-(4-Chlorophenyl)-4-((3-(trifluoromethyl)phenyl)selanyl)-1H-1,2,3-triazole* (**4f**): Yellow solid, mp: 45–47 °C. Yield: 0.091 g (45%). ^1^H NMR (400 MHz, CDCl_3_) δ: 8.05 (s, 1H), 7.69 (s, 1H), 7.63–7.60 (m, 3H), 7.44–7.41 (m, 3H), 7.29 (t, *J* = 7.8 Hz, 1H). ^13^C NMR (100 MHz, CDCl_3_) δ: 135.1, 135.0, 134.9, 132.5, 131.6 (q, *J*_C-F_ = 32.9 Hz), 131.3, 130.1, 129.7, 128.1 (q, *J*_C-F_ = 3.6 Hz), 126.8, 124.3 (q, *J*_C-F_ = 3.7 Hz), 123.5 (q, *J*_C-F_ = 272.7 Hz), 121.7. HRMS Calcd. for C_15_H_9_ClF_3_N_3_Se [M-N_2_ + H]^+^: 374.96431. Found: 374.9643.

*1-(4-Methoxyphenyl)-4-(phenylselanyl)-1H-1,2,3-triazole* (**4g**): [58] Light orange solid, mp: 70–72 °C. Yield: 0.136 g (82%). ^1^H NMR (400 MHz, CDCl_3_) δ: 7.91 (s, 1H), 7.53 (d, *J* = 8.8 Hz, 2H), 7.43–7.42 (m, 2H), 7.19–7.16 (m, 3H), 6.92 (d, *J* = 8.9 Hz, 2H), 3.77 (s, 3H). ^13^C NMR (100 MHz, CDCl_3_) δ: 160.0, 132.9, 132.6, 131.7, 129.3, 127.4, 126.7, 124.8, 122.1, 114.8, 55.6.

*1-(4-Fluorophenyl)-4-(phenylselanyl)-1H-1,2,3-triazole* (**4h**): white solid, mp: 78–80 °C. Yield: 0.126 g (79%). ^1^H NMR (300 MHz, CDCl_3_) δ: 8.02 (s, 1H), 7.72–7.68 (m, 2H), 7.54–7.51 (m, 2H), 7.26–7.24 (m, 3H), 7.22–7.19 (m, 2H). ^13^C NMR (75 MHz, CDCl_3_) δ: 162.5 (d, *J*_C-F_ = 249.6 Hz), 133.4, 132.8 (d, *J*_C-F_ = 3.4 Hz), 131.9, 130.0, 129.4, 127.5, 126.6, 122.5 (d, *J*_C-F_ = 8.7 Hz), 116.7 (d, *J*_C-F_ = 23.2 Hz). HRMS Calcd. for C_14_H_10_FN_3_Se [M + H]^+^: 320.0097. Found: 320.0099.

*1-(4-Iodophenyl)-4-(phenylselanyl)-1H-1,2,3-triazole* (**4i**): white solid, mp: 124–126 °C. Yield: 0.164 g (77%). ^1^H NMR (300 MHz, CDCl_3_) δ: 8.05 (s, 1H), 7.83 (d, *J* = 8.7 Hz, 2H), 7.54–7.47 (m, 4H), 7.26–7.24 (m, 3H). ^13^C NMR (75 MHz, CDCl_3_) δ: 138.8, 136.1, 133.8, 131.9, 129.9, 129.4, 127.6, 126.1, 121.9, 94.0. HRMS Calcd. for C_14_H_10_IN_3_Se [M + H]^+^: 427.9157. Found: 427.9160.

*1-(2-Fluorophenyl)-4-(phenylselanyl)-1H-1,2,3-triazole* (**4j**): Yellow solid, mp: 60–62 °C. Yield: 0.089 g. (56%). ^1^H NMR (400 MHz, CDCl_3_) δ: 7.40–7.38 (m, 2H), 7.28–7.15 (m, 4H), 6.94–6.90 (m, 1H), 6.86–6.82 (m, 1H), 6.57–6.53 (m, 1H). ^13^C NMR (100 MHz, CDCl_3_) δ: 155.3 (d, *J*_C-F_ = 255.8 Hz), 136.4, 133.2, 132.6, 131.9 (d, *J*_C-F_ = 7.7 Hz), 129.3, 129.2, 128.8 (d, *J*_C-F_ = 23.9 Hz), 127.9, 126.9, 124.8, 117.0 (d, *J*_C-F_ = 19.2 Hz). HRMS Calcd. for C_14_H_10_FN_3_Se [M + H]^+^: 320.0097. Found: 320.0097.

*1-(3-Nitrophenyl)-4-(phenylselanyl)-1H-1,2,3-triazole* (**4k**): Yellow solid, mp: 109–111 °C. Yield: 0.130 g (75%). ^1^H NMR (400 MHz, CDCl_3_) δ: 8.50 (s, 1H), 8.24 (d, *J* = 7.1 Hz, 1H), 8.11–8.08 (m, 2H), 7.68 (d, *J* = 8.1 Hz, 1H), 7.50–7.49 (m, 2H), 7.21–7.19 (m, 3H). ^13^C NMR (100 MHz, CDCl_3_) δ: 148.9, 137.3, 134.9, 132.4, 131.1, 129.5, 127.9, 126.0 (2C), 125.9, 123.4, 115.2. HRMS Calcd. for C_14_H_10_N_4_O_2_Se [M-N_2_ + H]^+^: 318.9981. Found: 318.9979.

*1-Benzyl-4-(phenylselanyl)-1H-1,2,3-triazole* (**4l**): [56] White solid, mp: 54–56 °C. Yield: 0.113 g (72%). ^1^H NMR (400 MHz, CDCl_3_) δ: 7.48 (s, 1H), 7.35–7.33 (m, 2H), 7.29–7.25 (m, 3H), 7.18–7.17 (m, 2H), 7.13–7.10 (m, 3H), 5.45 (s, 2H). ^13^C NMR (100 MHz, CDCl_3_) δ: 134.1, 132.5, 131.3, 130.6, 129.2, 129.1, 128.9, 128.4, 128.1, 127.2, 54.3.

*7-Chloro-4-(4-(phenylselanyl)-1H-1,2,3-triazol-1-yl)quinoline* (**4m**): Orange solid, mp: 48–50 °C. Yield: 0.154 g (80%). ^1^H NMR (400 MHz, CDCl_3_) δ: 8.93 (d, *J* = 4.6 Hz, 1H), 8.12 (d, *J* = 2.1 Hz, 1H), 8.01 (s, 1H), 7.84 (d, *J* = 9.1 Hz, 1H), 7.52–7.47 (m, 3H), 7.37 (d, *J* = 4.6 Hz, 1H), 7.21–7.18 (m, 3H). ^13^C NMR (100 MHz, CDCl_3_) δ: 151.3, 150.1, 140.4, 136.9, 134.1, 132.4, 129.5 (2C), 129.4, 129.1, 128.9, 127.9, 124.3, 120.3, 115.9. HRMS Calcd. For C_17_H_12_ClN_4_Se [M + H]^+^: 386.9916. Found: 386.9921.

*1-(5-Hidroxymethyl)-4-(4-phenylselanyl)-1H-1,2,3-triazo-1-yl)tetrahydrofuran-2-yl)-5-methylpyrimidine-2,4(1H, 3H) dione* (**4n**): Yield: 0.108 g (48%); White solid; mp 101–103 °C; ^1^H NMR (CDCl_3_, 400 MHz): δ 11.36 (s, 1H), 8.67 (s, 1H), 7.82 (s, 1H), 7.37 (d, *J* = 9.0 Hz, 2H), 7.32–7.24 (m, 3H), 6.43 (t, *J* = 6.6 Hz, 1H), 5.45–5.40 (m, 1H), 5,28 (t, *J* = 5.2 Hz, 1H), 4.25 (q, *J* = 3.5 Hz, 1H), 3.74–3.62 (m, 2H), 2.82–2.63 (m, 2H), 1.81 (s, 3H). ^13^C NMR (CDCl_3_, 100 MHz): δ 163.7; 150.4; 136.2; 130.7; 130.2; 130.1; 129.9; 129.5; 127.0; 109.6; 84.3; 83.9; 60.7; 59.6; 37.0; 12.2. HRMS Calcd. For C_18_H_20_N_5_O_4_Se [M + H]^+^: 450.0676. Found: 450.0673.

*1-(4-Chlorophenyl)-4-(phenylselanyl)-5-((phenylselanyl)ethynyl)-1H-1,2,3-triazole* (**5a**): White solid, mp: 71–73 °C. Yield: 0.043 g (17%). ^1^H NMR (400 MHz, CDCl_3_) δ: 7.71 (d, *J* = 8.9 Hz, 2H), 7.60–7.58 (m, 2H), 7.47–7.43 (m, 4H), 7.33–7.25 (m, 6H). ^13^C NMR (100 MHz, CDCl_3_) δ: 138.0, 135.6, 134.9, 133.1, 130.3, 130.0, 129.7, 129.5, 129.1, 128.2, 128.0, 127.0, 125.2, 124.6, 87.5, 87.5. ^77^Se NMR (76 MHz, CDCl_3_) δ: 301.52, 298.40. HRMS Calcd. for C_22_H_14_ClN_3_Se_2_: [M + H]^+^: 515.9279. Found: 515.9275.

*1-(4-Fluorophenyl)-4-(phenylselanyl)-5-((phenylselanyl)ethynyl)-1H-1,2,3-triazole* (**5b**): White solid, mp: 67–69 °C. Yield: 0.037 g (15%). ^1^H NMR (400 MHz, CDCl_3_) δ: 7.72 (dd, *J* = 9.0 and 4.7 Hz, 2H), 7.61–7.57 (m, 2H), 7.47–7.44 (m, 2H), 7.30–7.24 (m, 6H), 7.16 (dd, *J* = 9.0 and 8.1 Hz, 2H). ^13^C NMR (100 MHz, CDCl_3_) δ: 162.9 (d, *J*_C-F_ = 250.4 Hz), 137.8, 133.0, 132.52 (d, *J*_C-F_ = 3.2 Hz), 130.2, 129.9, 129.4, 129.1, 128.1, 127.9, 127.0, 125.5 (d, *J*_C-F_ = 8.8 Hz), 125.3, 116.5 (d, *J*_C-F_ = 23.3 Hz), 87.5, 87.1. HRMS Calcd. for C_22_H_14_FN_3_Se_2_: [M + H]^+^: 499.9575. Found: 499.9582.

## 4. Conclusions

In summary, we have described a one-pot strategy to prepare 4-arylselanyl-1*H*-1,2,3-triazoles starting from easily prepared and bench-stable selanylalkynylcarbinols. The protocol involves the generation of the terminal selanyl alkynes in situ and afforded the expected selenium-containing triazoles in a selective and efficient way. The use of a one-pot protocol avoids the isolation and purification steps of the reactive terminal selanylalkynes. The strategy was successfully employed in the synthesis of selanyltriazole-functionalized chloroquine and Zidovudine. Further studies are ongoing to better characterize the pharmacological potential of these new compounds.

## Figures and Tables

**Table 1:** Optimization of the reaction conditions. ^a^

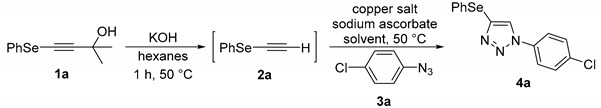			
**Entry**	**Copper Salt**	**Solvent**	**Yield (%) ^b^**
**1**	**Cu(OAc)_2_·H_2_O**	**THF/H_2_O**	**85**
2	CuI	THF/H_2_O	60
3 ^c^	CuI	DMSO	32
4	CuO_nps_	THF/H_2_O	traces
5	CuCl_2_	THF/H_2_O	75
6	Cu(OAc)_2_·H_2_O	THF	-
7 ^d^	Cu(OAc)_2_·H_2_O	THF/H_2_O	40
8 ^e^	Cu(OAc)_2_·H_2_O	THF/H_2_O	65
9 ^f^	Cu(OAc)_2_·H_2_O	THF/H_2_O	40
10 ^g^	Cu(OAc)_2_·H_2_O	THF/H_2_O	50

^a^ General reaction conditions: Compound **1a** (1 mmol) was subjected to the retro-Favorskii reaction [58]. After its completion (followed by TLC), azide **3a** (0.5 equiv) was added, followed by sodium ascorbate (10 mol%), the copper salt (5 mol%), THF (0.5 mL) and H_2_O (0.5 mL). The resulting mixture was stirred for 8 h at 50 °C. ^b^ Yields of isolated product **4a**. ^c^ Reaction performed in the absence of sodium ascorbate and in the presence of Et_3_N (1 equiv). ^d^ Reaction performed using 3 mol% of Cu(OAc)_2_·H_2_O and 6 mol% of sodium ascorbate. ^e^ Argon atmosphere was employed. ^f^ Reaction performed at room temperature. ^g^ Reaction performed using 1 equiv. of azide **3a**.

## Data Availability

Data supporting the reported results can be found with the main author (D.A.).

## Supplementary Materials

The following are available online, ^1^H and ^13^C NMR spectra of all compounds.

## Sample Availability

Samples of the compounds **4a**-**n** are available from the authors.
