# Supplementary material for: Synthesis of 4-Arylselanyl-1H-1,2,3-triazoles from Selenium-Containing Carbinols
Source: Molecules. 2021 Apr 12;26(8):2224. doi: 10.3390/molecules26082224 (PMC8070154; doi:10.3390/molecules26082224)
Supplement: Supplementary file 1 [file molecules-26-02224-s001.pdf]

molecules

Synthesis of 4-Arylselanyl-1*H*-1,2,3-triazoles from Selenium-Containing Carbinols

Francesca Begini [1], Renata A. Balaguez [2], Allya Larroza [2], Eric F. Lopes [2], Eder João Lenardão [2], Claudio Santi [1] and Diego Alves *,[2]

[1] Group of Catalysis, Synthesis and Organic Green Chemistry, Department of Pharmaceutical Sciences University of Perugia Via del Liceo 1, 06123 Perugia, Italy
[2] LASOL-CCQFA, Universidade Federal de Pelotas-UFPel, P.O. Box 354, 96010-900 Pelotas, RS, Brazil
* Correspondence: diego.alves@ufpel.edu.br; Tel.: +55-53-32757533

Contents

1. *General Remarks*…...S2

2. *General procedure for the synthesis of 4-arylselanyl-1H-1,2,3-triazoles 4*..................S2

3. *Spectral data of the products*..S3

4. *Spectra of the compounds*...S8

1. General remarks:

Reactions were carried out in a two-necked round-bottomed flask with Teflon-coated magnetic stirring bar. Solvents and reagents were used as received unless otherwise noted. The reactions were monitored by TLC performed by using Merck silica gel (60 F254), 0.25 mm thickness. For visualization, TLC plates were either placed under UV light, or stained with iodine vapor or 5% vanillin in 10% H_2SO_4 under heating. Column chromatography was performed by using Merck silica gel (230–400 mesh). Carbon-13 nuclear magnetic resonance spectra (^{13}C NMR) were obtained at 75 MHz on Bruker DPX 300 spectrometer and at 100 MHz on Bruker Avance III HD 400 spectrometer. Spectra were recorded in $CDCl_3$ solutions. Chemical shifts are reported in ppm, referenced to tetramethylsilane (TMS) as the external reference (^1H NMR) or to the solvent peak of $CDCl_3$ (^{13}C NMR). Coupling constants (*J*) are reported in Hertz. Abbreviations to denote the multiplicity of a particular signal are s (singlet), d (doublet), t (triplet), dd (double doublet), q (quartet) and m (multiplet). High resolution mass spectra (HRMS) were recorded on a Bruker Micro TOF-QII spectrometer 10416. Reagents 2-methyl-3-butyn-2-ol and selenium powder were purchased from Sigma-Aldrich. The starting materials selanylalkynyl-carbinols were synthesized according to the previous literature [1].

2. General procedure for the synthesis of 4-arylselanyl-1H-1,2,3-triazoles 4:

The arylselanyl carbinol 1 (1.0 mmol), KOH (1.1 mmol, 0.062 g), and hexanes (3.0 mL) were added to a 25 mL two-necked round-bottomed flask equipped with reflux condenser. The system was then immersed in a preheated oil bath at 50 °C and stirred at this temperature for 1 to 5 hours.[1] Then, 0.5 mmol of the appropriate azide 3, $Cu(OAc)_2·H_2O$ (0.025 mmol), sodium ascorbate (0.5 mmol), THF (0.5 mL) and H_2O (0.5 mL) were added to the reaction flask. The resulting solution was stirred at 50 °C for 8 hours. Then, a saturated solution of NH_4Cl (10 mL) was added, followed by the addition of EtOAc (10 mL). The organic layer was separated and the aqueous phase was extracted with EtOAc (3x 10 mL), dried over $MgSO_4$, and the solvent was evaporated under reduced pressure. The crude product was purified by column chromatography on silica gel with a mixture of

hexane/ethyl acetate (9:1) as eluent. Spectral data for the prepared products are listed below.

3. Spectral data of the products:

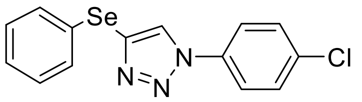

1-(4-chlorophenyl)-4-(phenylselanyl)-1H-1,2,3-triazole 4a: Pale yellow solid, mp: 105-107 °C. Yield: 0.142 g (85%). ^{1}H NMR (300 MHz, $CDCl_3$) δ: 8.05 (s, 1H), 7.67 (d, *J* = 8.9 Hz, 2H), 7.54 – 7.47 (m, 4H), 7.26 – 7.24 (m, 3H). ^{13}C NMR (75 MHz, $CDCl_3$) δ: 135.0, 134.8, 133.7, 131.9, 129.9, 129.4, 127.6, 126.3, 124.4, 121.6. HRMS Calcd. for $C_{14}H_{10}ClN_3Se$ $[M+H]^+$: 335.9799. Found: 335.9802.

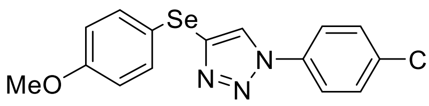

1-(4-chlorophenyl)-4-((4-methoxyphenyl)selanyl)-1H-1,2,3-triazole 4b: Yellow solid, mp: 86-88 °C. Yield: 0.137 g (75 %). ^{1}H NMR (400 MHz, $CDCl_3$) δ: 7.92 (s, 1H), 7.64 (d, *J* = 8.9 Hz, 2H), 7.57 (d, *J* = 8.8 Hz, 2H), 7.47 (d, *J* = 8.9 Hz, 1H), 6.82 (d, *J* = 8.8 Hz, 2H), 3.78 (s, 3H). ^{13}C NMR (100 MHz, $CDCl_3$) δ: 159.8, 135.4, 135.2, 134.7, 129.9, 125.1, 124.3, 121.6, 119.3, 115.1, 55.3. HRMS Calcd. for $C_{15}H_{12}ClN_3OSe$ $[M-N_2+H]^+$: 337.9843. Found: 337.9843.

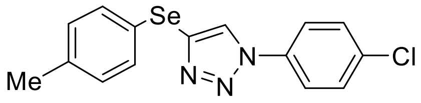

1-(4-chlorophenyl)-4-(*p*-tolylselanyl)-1H-1,2,3-triazole 4c: Yellow solid, mp: 74-75 °C. Yield: 0.115 g (66%). ^{1}H NMR (400 MHz, $CDCl_3$) δ: 7.87 (s, 1H), 7.58 (d, *J* = 8.9 Hz, 2H), 7.42 - 7.39 (m, 4H), 7.01 (d, *J* = 7.9 Hz, 2H). ^{13}C NMR (100 MHz, $CDCl_3$) δ: 137.9, 133.7, 132.8, 130.8, 130.2, 130.0, 129.5, 125.6, 124.4, 121.6, 21.0. HRMS Calcd. for $C_{15}H_{12}ClN_3Se$ $[M-N_2+H]^+$: 321.9894. Found: 321.9875.

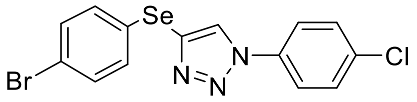

4-((4-bromophenyl)selanyl)-1-(4-chlorophenyl)-1H-1,2,3-triazole 4d: Yellow solid, mp: 46-48 °C. Yield: 0.122 g (59%). ^{1}H NMR (400 MHz, $CDCl_3$) δ: 7.99 (s, 1H), 7.61 (d, *J* = 8.9 Hz, 2H), 7.43 (d, *J* = 8.9 Hz, 2H), 7.33 - 7.28 (m, 4H). ^{13}C NMR (100 MHz, $CDCl_3$) δ: 135.0, 133.5, 132.4, 132.0, 130.0, 129.4, 128.9, 127.6, 126.4, 121.7. HRMS Calcd. for $C_{14}H_9BrClN_3Se$ $[M-N_2+H]^+$: 385.8840. Found: 385.8838.

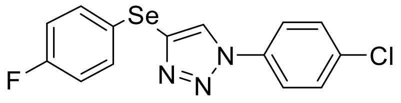

1-(4-chlorophenyl)-4-((4-fluorophenyl)selanyl)-1H-1,2,3-triazole 4e: Yellow solid, mp: 48-50 °C. Yield: 0.097 g (55%). ^{1}H NMR (400 MHz, $CDCl_3$) δ: 8.02 (s, 1H), 7.67 (d, *J* = 8.7 Hz, 2H), 7.56 (dd, *J* = 8.6 and 5.3 Hz, 2H), 7.49 (d, *J* = 8.7 Hz, 2H), 6.97 (t, *J* = 8.6 Hz, 2H). ^{13}C NMR (100 MHz, $CDCl_3$) δ: 162.6 (d, J_{C-F} = 248.1 Hz), 135.0, 134.9, 134.7 (d, J_{C-F} = 8.0 Hz), 131.1, 130.0, 125.9, 124.1 (d, J_{C-F} = 3.5 Hz), 121.6, 116.6 (d, J_{C-F} = 21.6 Hz). HRMS Calcd. for $C_{14}H_9ClFN_3Se$ $[M-N_2+H]^+$: 325.9643. Found: 325.9636.

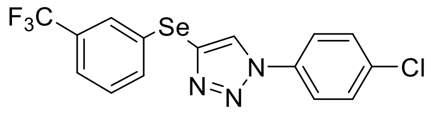

1-(4-chlorophenyl)-4-((3-(trifluoromethyl)phenyl)selanyl)-1H-1,2,3-triazole 4f: Yellow solid, mp: 45-47 °C. Yield: 0.091 g (45%). ^{1}H NMR (400 MHz, $CDCl_3$) δ: 8.05 (s, 1H), 7.69 (s, 1H), 7.63 - 7.60 (m, 3H), 7.44 - 7.41 (m, 3H), 7.29 (t, *J* = 7.8 Hz, 1H). ^{13}C NMR (100 MHz, $CDCl_3$) δ: 135.1, 135.0, 134.9, 132.5, 131.6 (q, $J_{C\text{-}F}$ = 32.9 Hz), 131.3, 130.1, 129.7, 128.1 (q, $J_{C\text{-}F}$ = 3.6 Hz), 126.8, 124.3 (q, $J_{C\text{-}F}$ = 3.7 Hz), 123.5 (q, $J_{C\text{-}F}$ = 272.7 Hz), 121.7. HRMS Calcd. for $C_{15}H_9ClF_3N_3Se$ $[M\text{-}N_2\text{+}H]^+$: 374.96431. Found: 374.9643

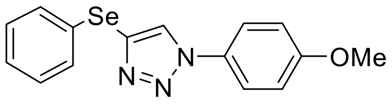

1-(4-methoxyphenyl)-4-(phenylselanyl)-1H-1,2,3-triazole [1] 4g: Light orange solid, mp: 70-72 °C. Yield: 0.136 g (82%). ^{1}H NMR (400 MHz, $CDCl_3$) δ: 7.91 (s, 1H), 7.53 (d, *J* = 8.8 Hz, 2H), 7.43 - 7.42 (m, 2H), 7.19 - 7.16 (m, 3H), 6.92 (d, *J* = 8.9 Hz, 2H), 3.77 (s, 3H). ^{13}C NMR (100 MHz, $CDCl_3$) δ: 160.0, 132.9, 132.6, 131.7, 129.3, 127.4, 126.7, 124.8, 122.1, 114.8, 55.6.

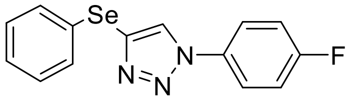

1-(4-fluorophenyl)-4-(phenylselanyl)-1H-1,2,3-triazole 4h: white solid, mp: 78-80 °C. Yield: 0.126 g (79%). ^{1}H NMR (300 MHz, $CDCl_3$) δ: 8.02 (s, 1H), 7.72 – 7.68 (m, 2H), 7.54 – 7.51 (m, 2H), 7.26 – 7.24 (m, 3H), 7.22 – 7.19 (m, 2H). ^{13}C NMR (75 MHz, $CDCl_3$) δ: 162.5 (d, $J_{C\text{-}F}$ = 249.6 Hz), 133.4, 132.8 (d, $J_{C\text{-}F}$ = 3.4 Hz), 131.9, 130.0, 129.4, 127.5, 126.6, 122.5 (d, $J_{C\text{-}F}$ = 8.7 Hz), 116.7 (d, $J_{C\text{-}F}$ = 23.2 Hz). HRMS Calcd. for $C_{14}H_{10}FN_3Se$ $[M\text{+}H]^+$: 320.0097. Found: 320.0099.

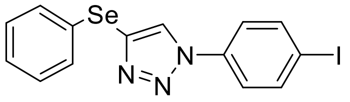

1-(4-iodophenyl)-4-(phenylselanyl)-1H-1,2,3-triazole 4i: white solid, mp: 124-126 °C. Yield: 0.164 g (77%). ^{1}H NMR (300 MHz, $CDCl_3$) δ: 8.05 (s, 1H), 7.83 (d, *J* = 8.7 Hz, 2H), 7.54 – 7.47 (m, 4H), 7.26 – 7.24 (m, 3H). ^{13}C NMR (75 MHz, $CDCl_3$) δ: 138.8, 136.1, 133.8, 131.9, 129.9, 129.4, 127.6, 126.1, 121.9, 94.0. HRMS Calcd. for $C_{14}H_{10}IN_3Se$ $[M\text{+}H]^+$: 427.9157. Found: 427.9160.

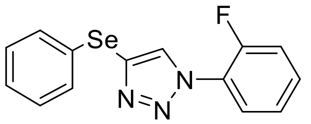

1-(2-fluorophenyl)-4-(phenylselanyl)-1H-1,2,3-triazole 4j: Yellow solid, mp: 60-62 °C. Yield: 0.089 g. (56%). ^{1}H NMR (400 MHz, $CDCl_3$) δ: 7.40 - 7.38 (m, 2H), 7.28 - 7.15 (m, 4H), 6.94 - 6.90 (m, 1H), 6.86 - 6.82 (m, 1H), 6.57 - 6.53 (m, 1H). ^{13}C NMR (100 MHz, $CDCl_3$) δ: 155.3 (d, $J_{C\text{-}F}$ = 255.8 Hz), 136.4, 133.2, 132.6, 131.9 (d, $J_{C\text{-}F}$ = 7.7 Hz), 129.3, 129.2, 128.8 (d, $J_{C\text{-}F}$ = 23.9 Hz), 127.9, 126.9, 124.8, 117.0 (d, $J_{C\text{-}F}$ = 19.2 Hz). HRMS Calcd. for $C_{14}H_{10}FN_3Se$ $[M\text{+}H]^+$: 320.0097. Found: 320.0097.

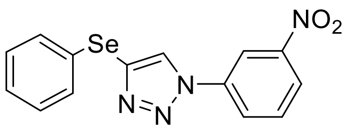

1-(3-nitrophenyl)-4-(phenylselanyl)-1H-1,2,3-triazole 4k: Yellow solid, mp: 109-111 °C. Yield: 0.130 g (75%). ^{1}H NMR (400 MHz, $CDCl_3$) δ: 8.50 (s, 1H), 8.24 (d, *J* = 7.1 Hz, 1H), 8.11 - 8.08 (m, 2H), 7.68 (d, *J* = 8.1 Hz, 1H), 7.50 - 7.49 (m, 2H), 7.21 - 7.19 (m, 3H). ^{13}C NMR (100 MHz, $CDCl_3$) δ: 148.9, 137.3, 134.9, 132.4, 131.1, 129.5, 127.9, 126.0 (2C), 125.9, 123.4, 115.2. HRMS Calcd. for $C_{14}H_{10}N_4O_2Se$ [M-N_2+H]$^+$: 318.9981. Found: 318.9979.

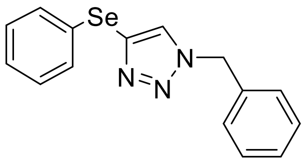

1-benzyl-4-(phenylselanyl)-1H-1,2,3-triazole [2] 4l: White solid, mp: 54-56 °C. Yield: 0.113 g (72%). ^{1}H NMR (400 MHz, $CDCl_3$) δ: 7.48 (s, 1H), 7.35 - 7.33 (m, 2H), 7.29 - 7.25 (m, 3H), 7.18 - 7.17 (m, 2H), 7.13 - 7.10 (m, 3H), 5.45 (s, 2H). ^{13}C NMR (100 MHz, $CDCl_3$) δ: 134.1, 132.5, 131.3, 130.6, 129.2, 129.1, 128.9, 128.4, 128.1, 127.2, 54.3.

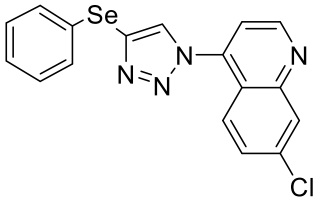

7-chloro-4-(4-(phenylselanyl)-1H-1,2,3-triazol-1-yl)quinoline 4m: Orange solid, mp: 48-50 °C. Yield: 0.154 g (80%). ^{1}H NMR (400 MHz, $CDCl_3$) δ: 8.93 (d, *J* = 4.6 Hz, 1H), 8.12 (d, *J* = 2.1 Hz, 1H), 8.01 (s, 1H), 7.84 (d, *J* = 9.1 Hz, 1H), 7.52 - 7.47 (m, 3H), 7.37 (d, *J* = 4.6 Hz, 1H), 7.21 - 7.18 (m, 3H). ^{13}C NMR (100 MHz, $CDCl_3$) δ: 151.3, 150.1, 140.4, 136.9, 134.1, 132.4, 129.5 (2C), 129.4, 129.1, 128.9, 127.9, 124.3, 120.3, 115.9. HRMS Calcd. for $C_{17}H_{12}ClN_4Se$ [M+H]$^+$: 386.9916. Found: 386.9921.

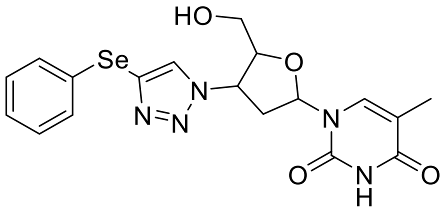

1-(5-hidroxymethyl)-4-(4-phenylselanyl)-1*H*-1,2,3-triazo-1-yl)tetrahydrofuran-2-yl)-5-methylpyrimidine-2,4(1*H*, 3*H*)-dione 4n: Yield: 0.108 g (48%); White solid; mp 101-103 °C; ^{1}H NMR ($CDCl_3$, 400 MHz): δ 11.36 (s, 1H), 8.67 (s, 1H), 7.82 (s, 1H), 7.37 (d, *J* = 9.0 Hz, 2H), 7.32-7.24 (m, 3H), 6.43 (t, *J* = 6.6 Hz, 1H), 5.45-5.40 (m, 1H), 5,28 (t, *J* = 5.2 Hz, 1H), 4.25 (q, *J* = 3.5 Hz, 1H), 3.74-3.62 (m, 2H), 2.82-2.63 (m, 2H), 1.81 (s, 3H). ^{13}C NMR ($CDCl_3$, 100 MHz): δ 163.7; 150.4; 136.2; 130.7; 130.2; 130.1; 129.9; 129.5; 127.0; 109.6; 84.3; 83.9; 60.7; 59.6; 37.0; 12.2. HRMS Calcd. for $C_{18}H_{20}N_5O_4Se$ [M+H]$^+$: 450.0676. Found: 450.0673.

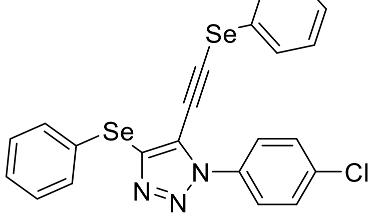

1-(4-chlorophenyl)-4-(phenylselanyl)-5-((phenylselanyl)ethynyl)-1H-1,2,3-triazole 5a: White solid, mp: 71-73 °C. Yield: 0.043 g (17%). ^{1}H NMR (400 MHz, $CDCl_3$) δ: 7.71 (d, *J* = 8.9 Hz, 2H), 7.60 – 7.58 (m, 2H), 7.47 – 7.43 (m, 4H), 7.33 – 7.25 (m, 6H). ^{13}C NMR (100 MHz, $CDCl_3$) δ: 138.0, 135.6, 134.9, 133.1, 130.3, 130.0, 129.7, 129.5, 129.1, 128.2, 128.0, 127.0,

125.2, 124.6, 87.5, 87.5. ^{77}Se NMR (76 MHz, $CDCl_3$) δ: 301.52, 298.40. HRMS Calcd. for $C_{22}H_{14}ClN_3Se_2$: $[M+H]^+$: 515.9279. Found: 515.9275.

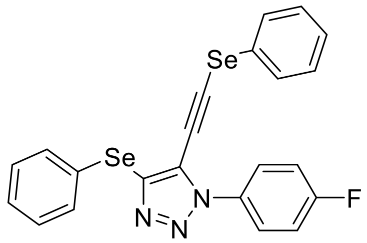

1-(4-fluorophenyl)-4-(phenylselanyl)-5-((phenylselanyl)ethynyl)-1H-1,2,3-triazole 5b: White solid, mp: 67-69 °C. Yield: 0.037 g (15%). ^{1}H NMR (400 MHz, $CDCl_3$) δ: 7.72 (dd, J = 9.0 and 4.7 Hz, 2H), 7.61 – 7.57 (m, 2H), 7.47 – 7.44 (m, 2H), 7.30 – 7.24 (m, 6H), 7.16 (dd, J = 9.0 and 8.1 Hz, 2H). ^{13}C NMR (100 MHz, $CDCl_3$) δ: 162.9 (d, J_{C-F} = 250.4 Hz), 137.8, 133.0, 132.52 (d, J_{C-F} = 3.2 Hz), 130.2, 129.9, 129.4, 129.1, 128.1, 127.9, 127.0, 125.5 (d, J_{C-F} = 8.8 Hz), 125.3, 116.5 (d, J_{C-F} = 23.3 Hz), 87.5, 87.1. HRMS Calcd. for $C_{22}H_{14}FN_3Se_2$: $[M+H]^+$: 499.9575. Found: 499.9582.

1. Lopes, E.F.; Dalberto, B.T.; Perin, G.; Alves, D.; Barcellos, T.; Lenardão, E.J. Synthesis of Terminal Ethynyl Aryl Selenides and Sulfides Based on the Retro-Favorskii Reaction of Hydroxypropargyl Precursors. *Chem. Eur. J.* **2017**, *23*, 13760–13765, doi:10.1002/chem.201702493.
2. Saraiva, M.; Seus, N.; de Souza, D.; Rodrigues, O.; Paixão, M.; Jacob, R.; Lenardão, E.; Perin, G.; Alves, D. Synthesis of [(Arylselanyl)Alkyl]-1,2,3-Triazoles by Copper-Catalyzed 1,3-Dipolar Cycloaddition of (Arylselanyl)Alkynes with Benzyl Azides. *Synthesis* **2012**, *44*, 1997–2004, doi:10.1055/s-0031-1291135.

4. Spectra of the compounds:

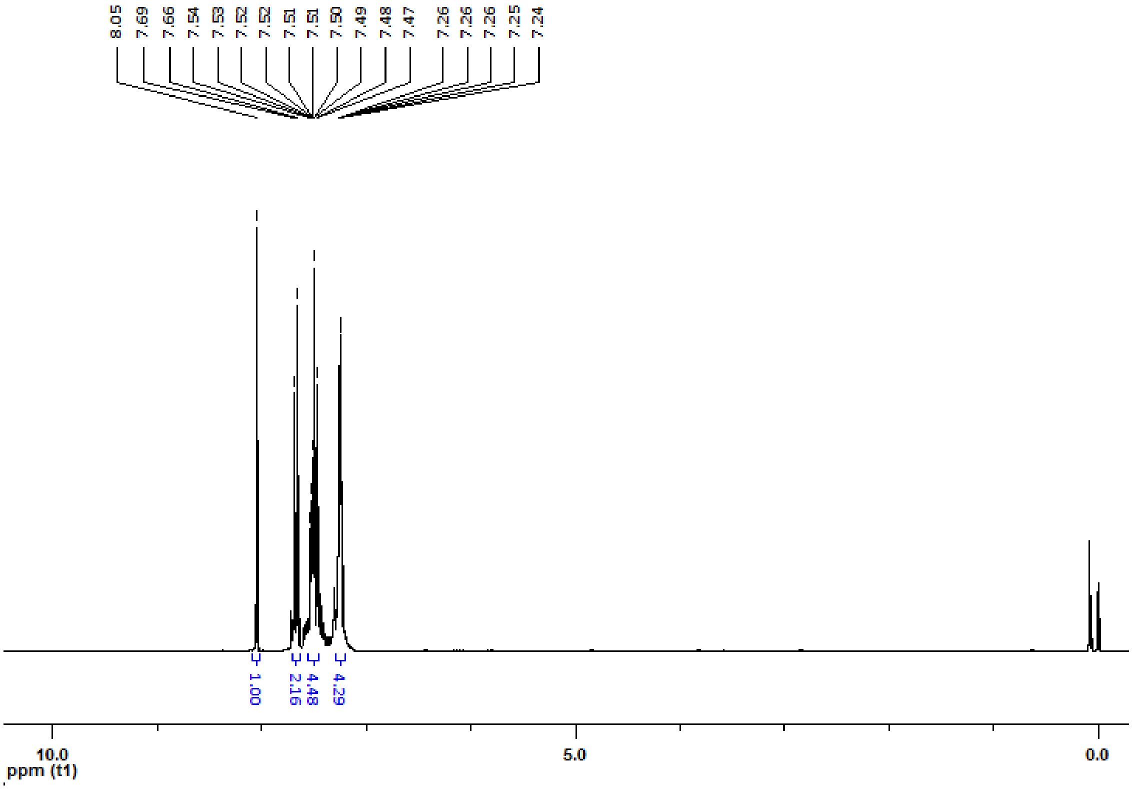

^{1}H NMR (300 MHz, $CDCl_3$) spectrum of **4a**.

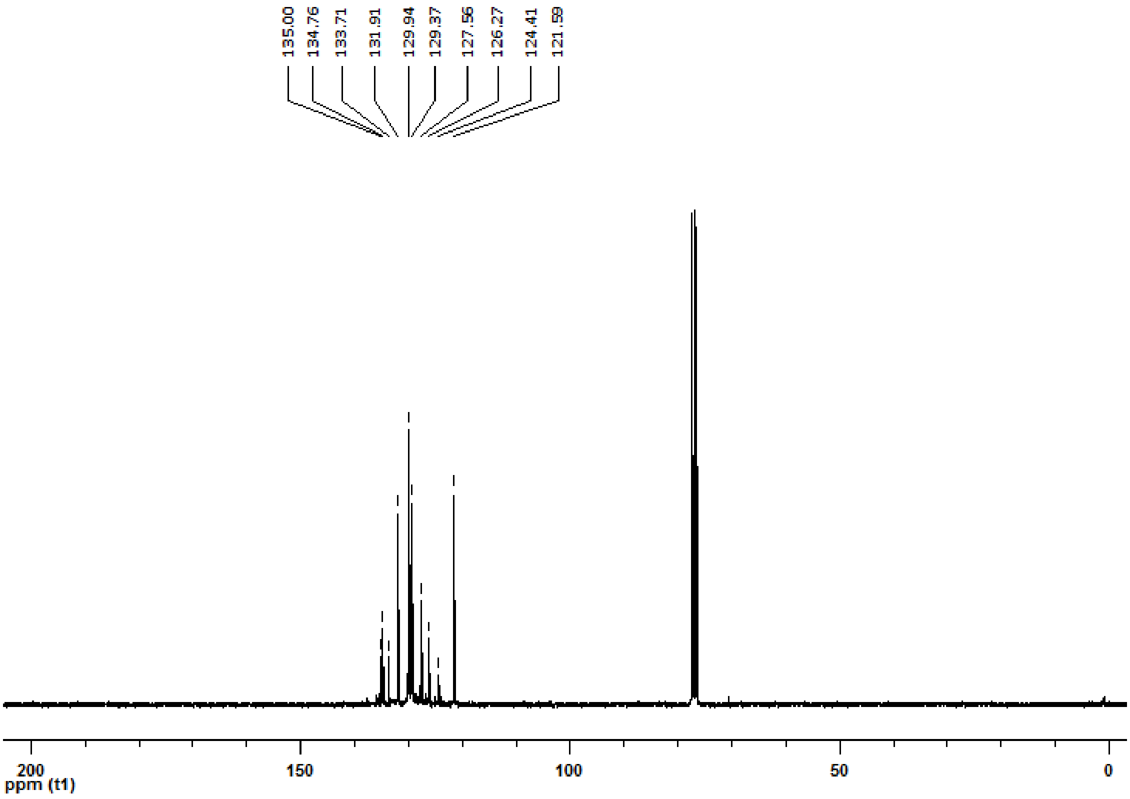

^{13}C NMR (75 MHz, $CDCl_3$) spectrum of **4a**.

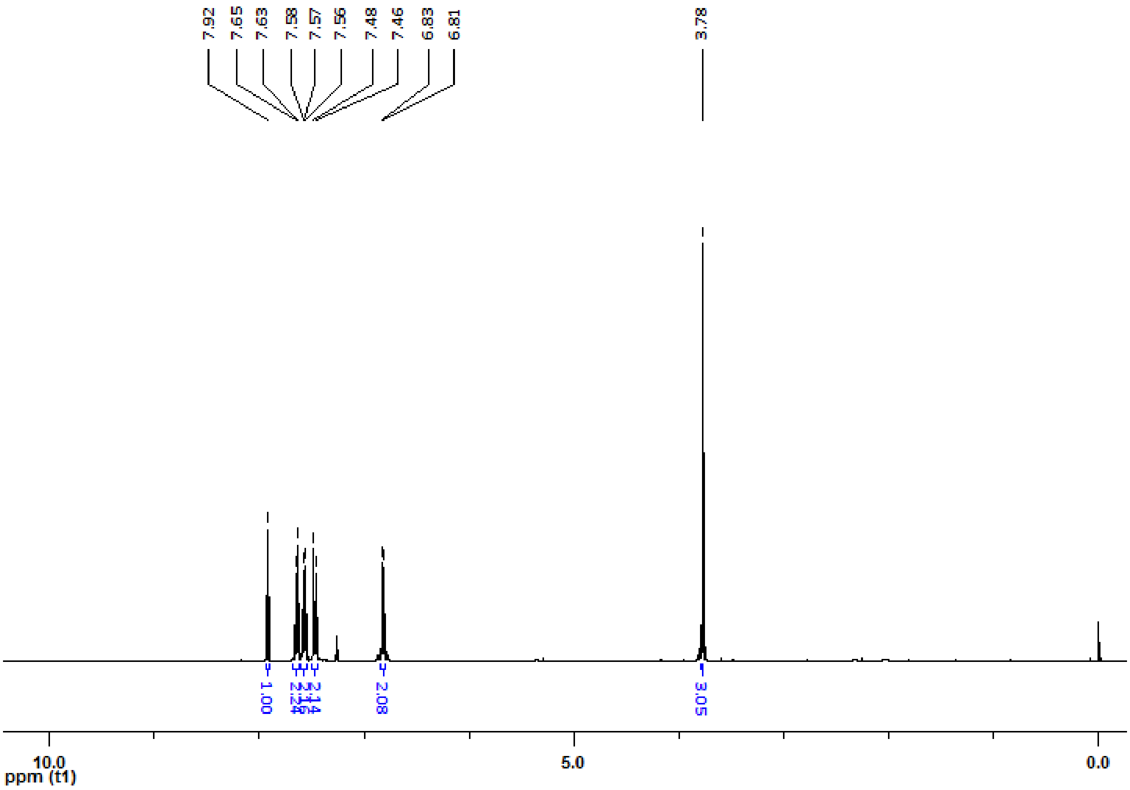

^{1}H NMR (400 MHz, $CDCl_3$) spectrum of **4b**.

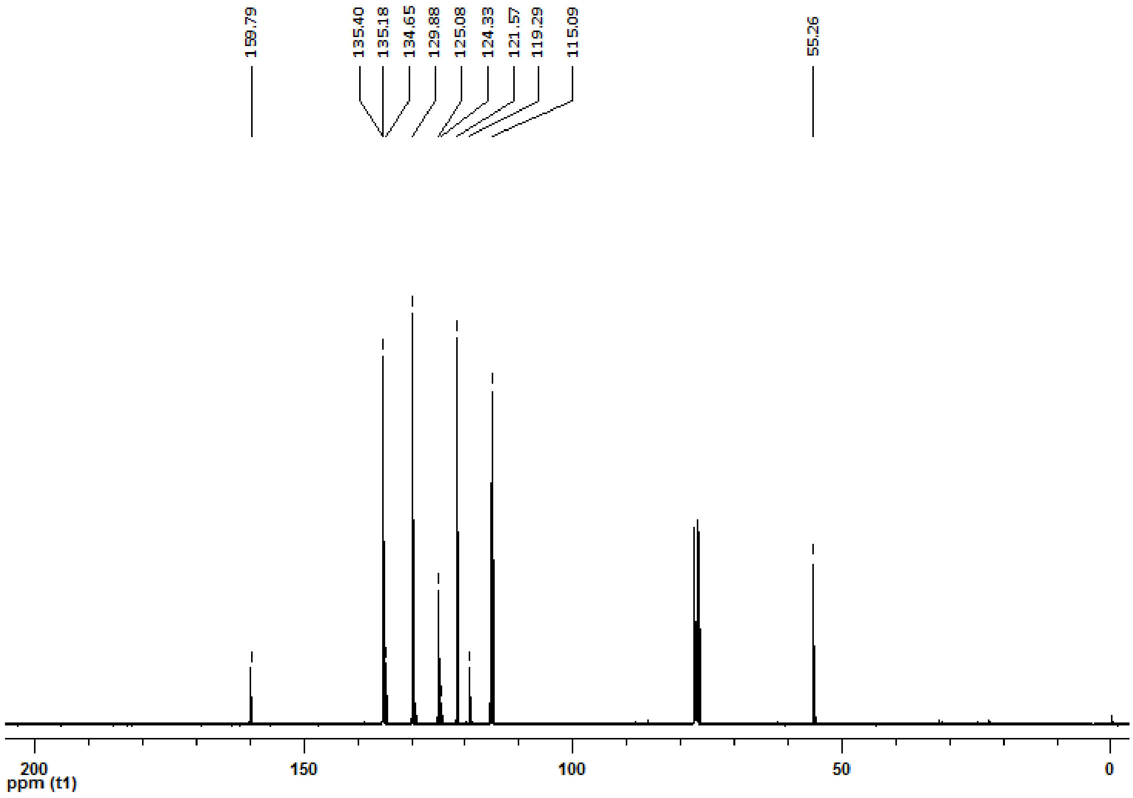

^{13}C NMR (100 MHz, $CDCl_3$) spectrum of **4b**.

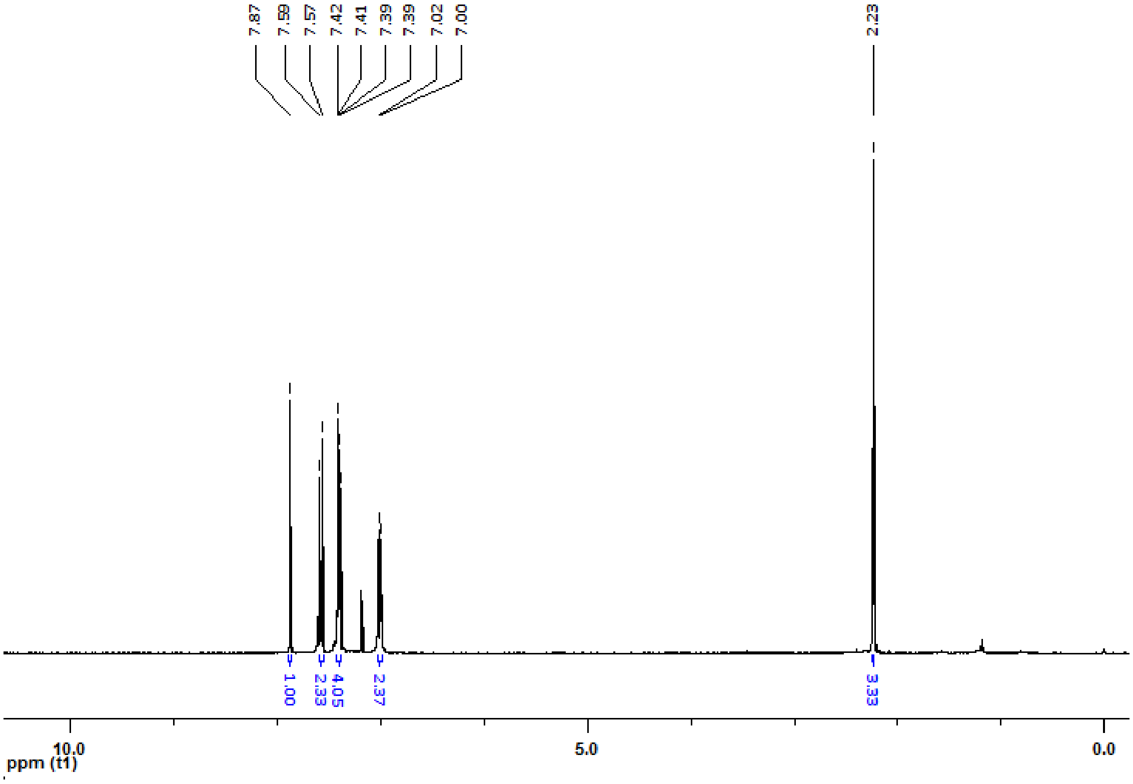

^{1}H NMR (400 MHz, $CDCl_3$) spectrum of **4c**.

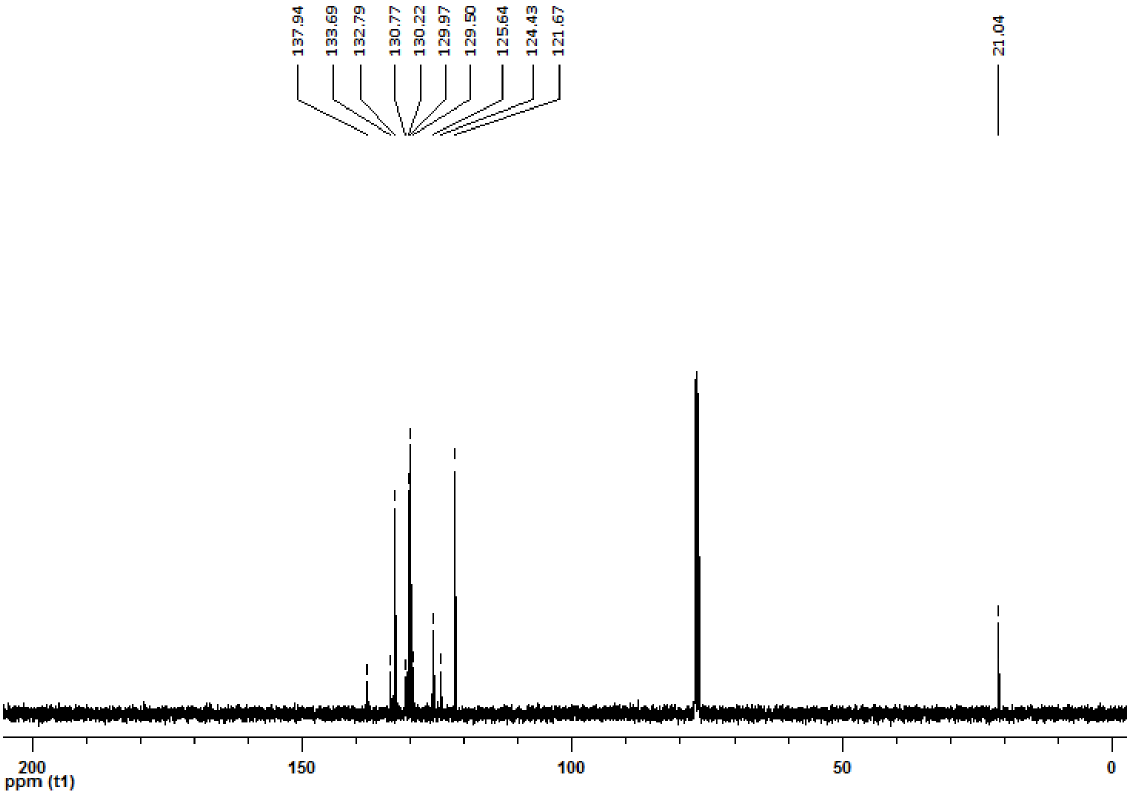

^{13}C NMR (100 MHz, $CDCl_3$) spectrum of **4c**.

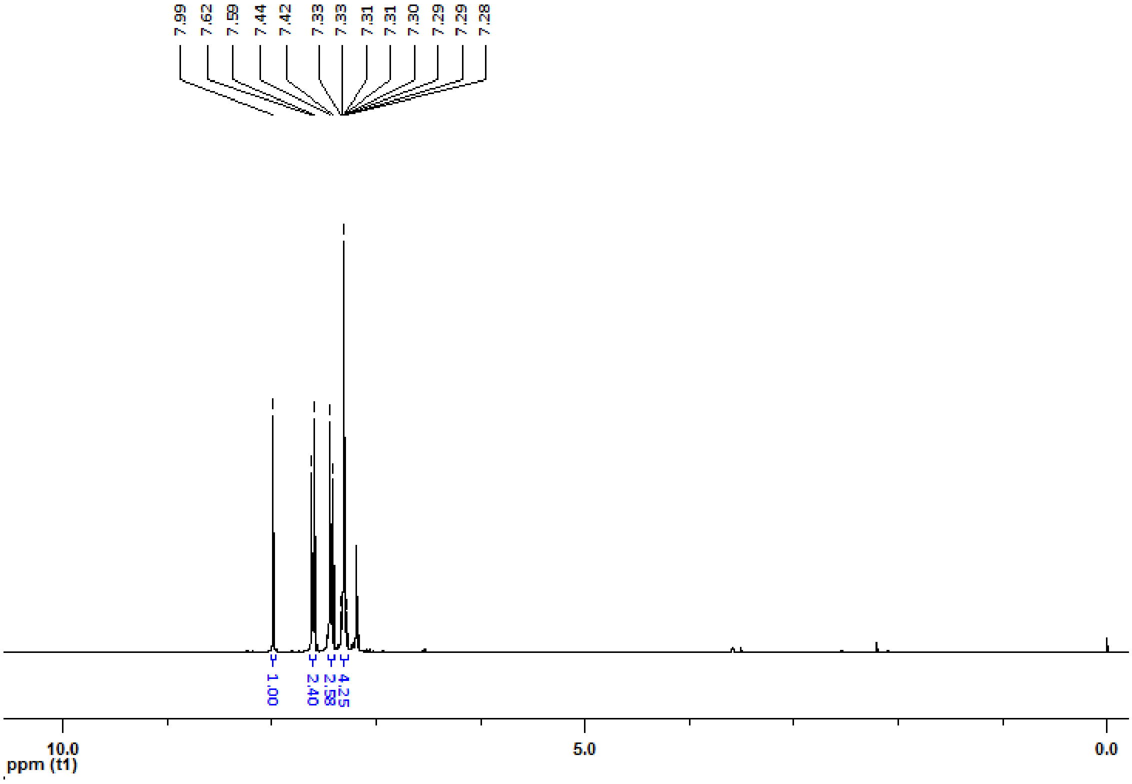

^{1}H NMR (400 MHz, $CDCl_3$) spectrum of **4d**.

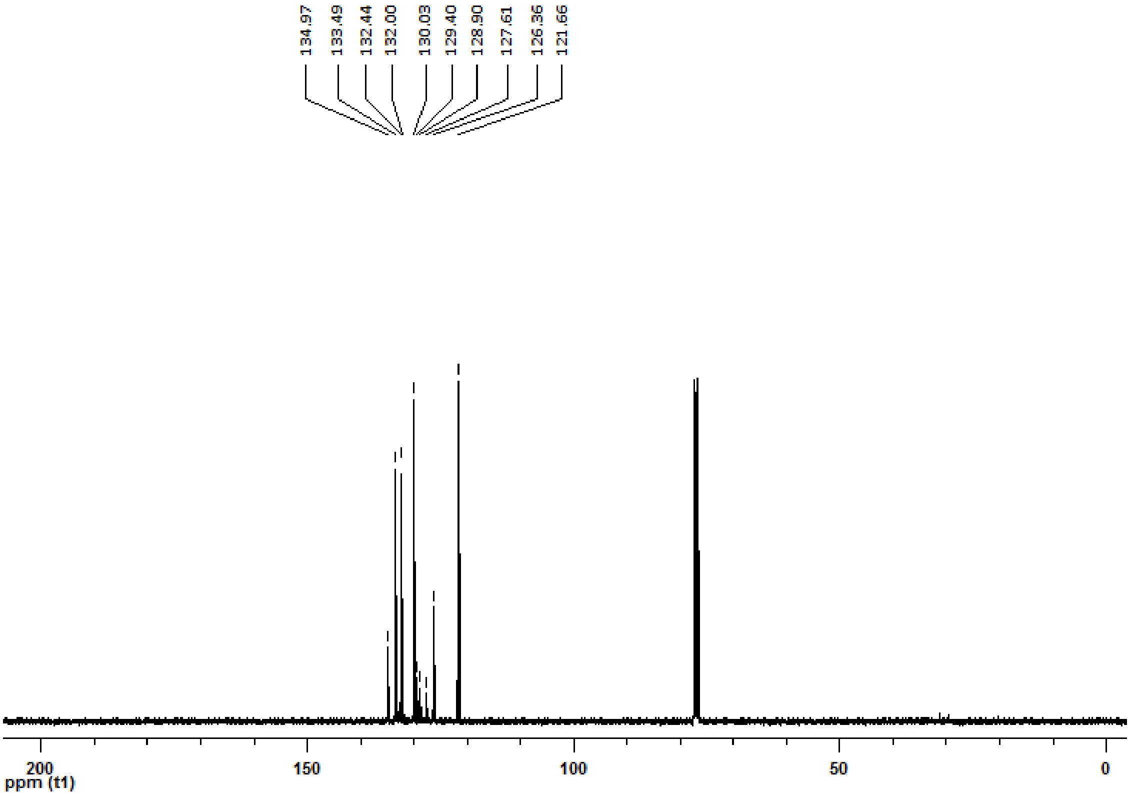

^{13}C NMR (100 MHz, $CDCl_3$) spectrum of **4d**.

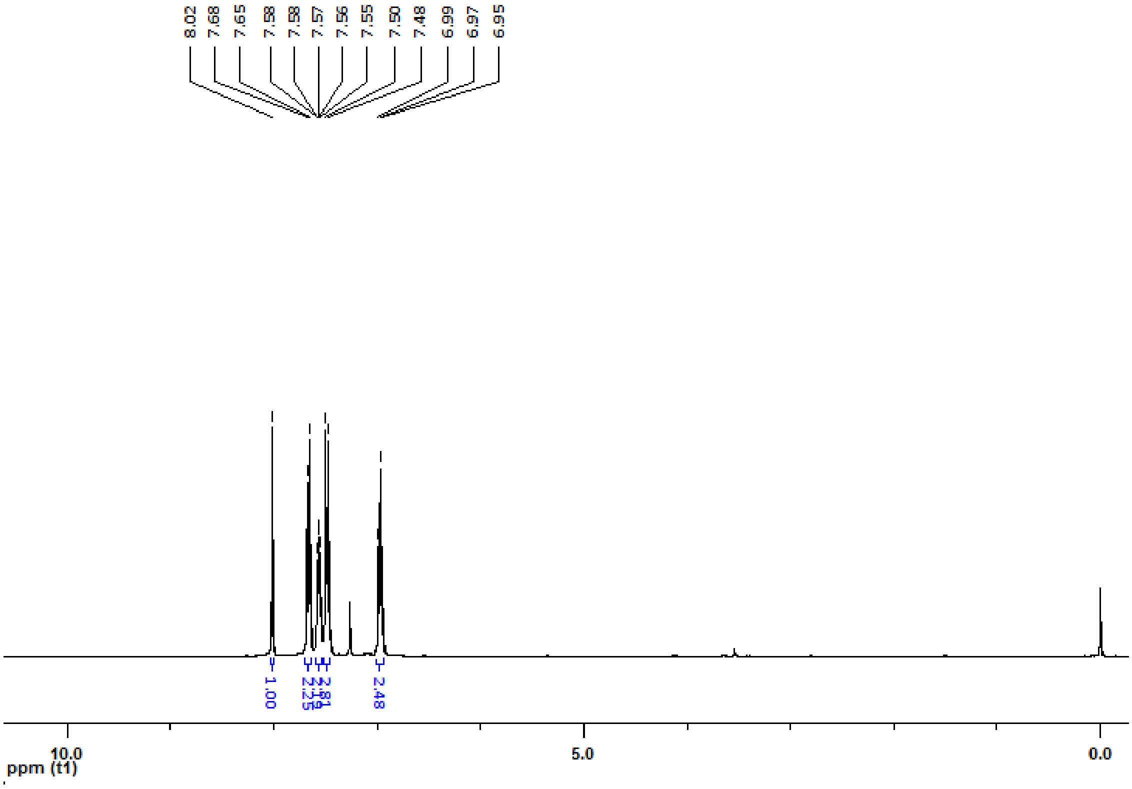

^{1}H NMR (400 MHz, $CDCl_3$) spectrum of **4e**.

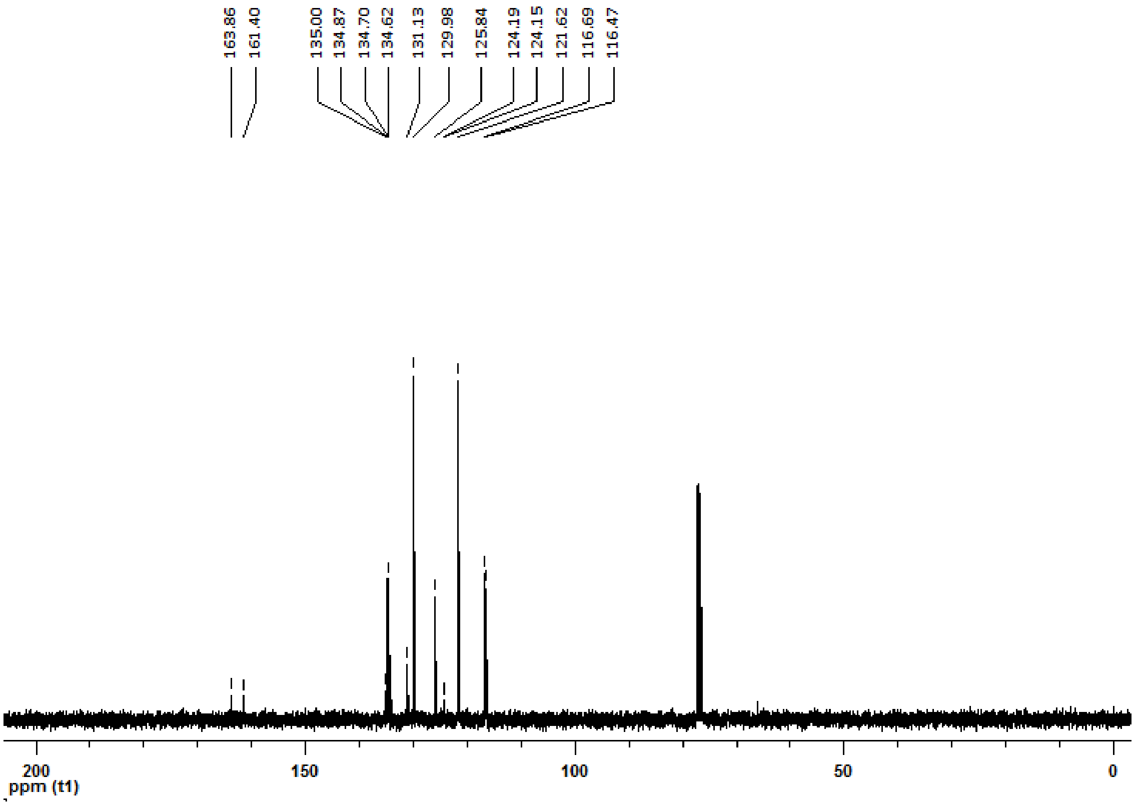

^{13}C NMR (100 MHz, $CDCl_3$) spectrum of **4e**.

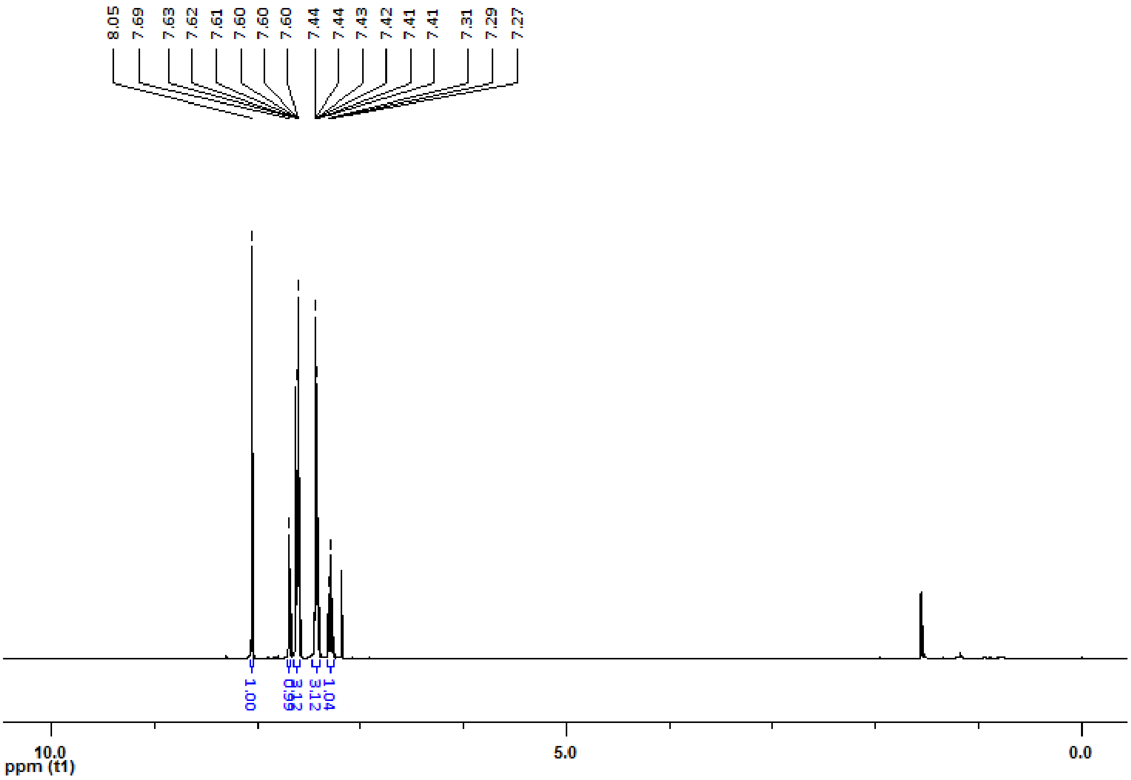

^{1}H NMR (400 MHz, $CDCl_3$) spectrum of **4f**.

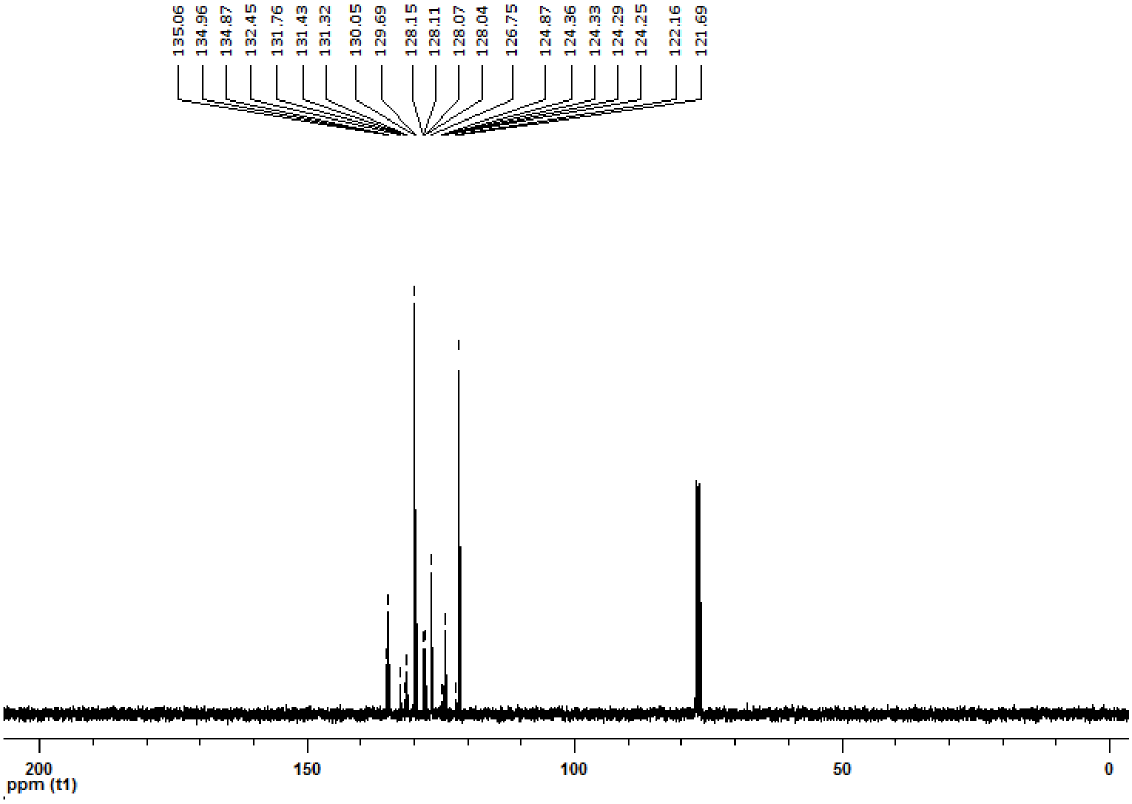

^{13}C NMR (100 MHz, $CDCl_3$) spectrum of **4f**.

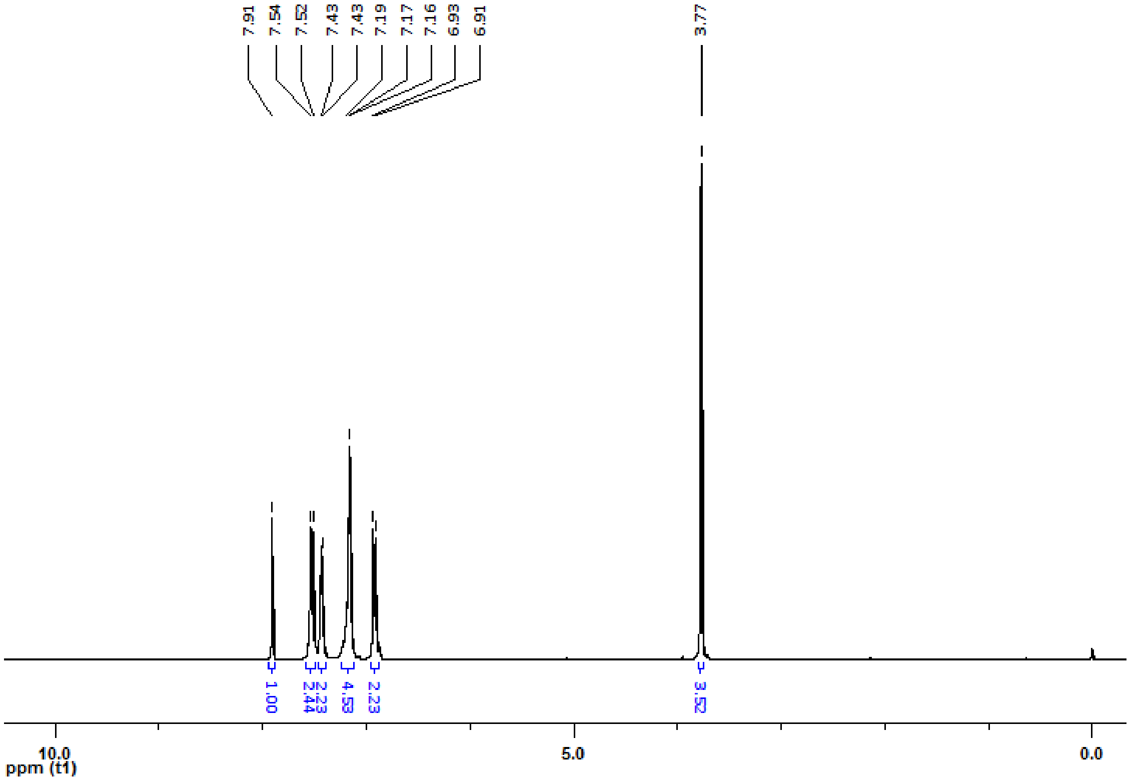

^{1}H NMR (400 MHz, $CDCl_3$) spectrum of **4g**.

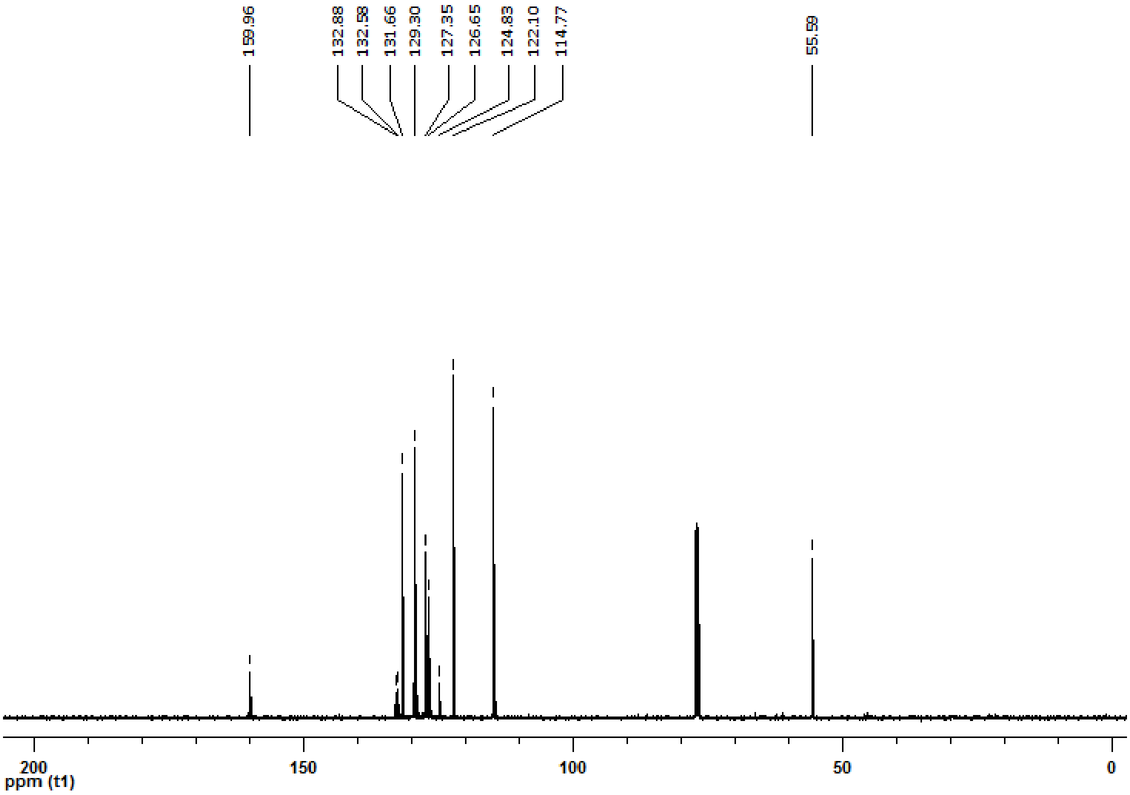

^{13}C NMR (100 MHz, $CDCl_3$) spectrum of **4g**.

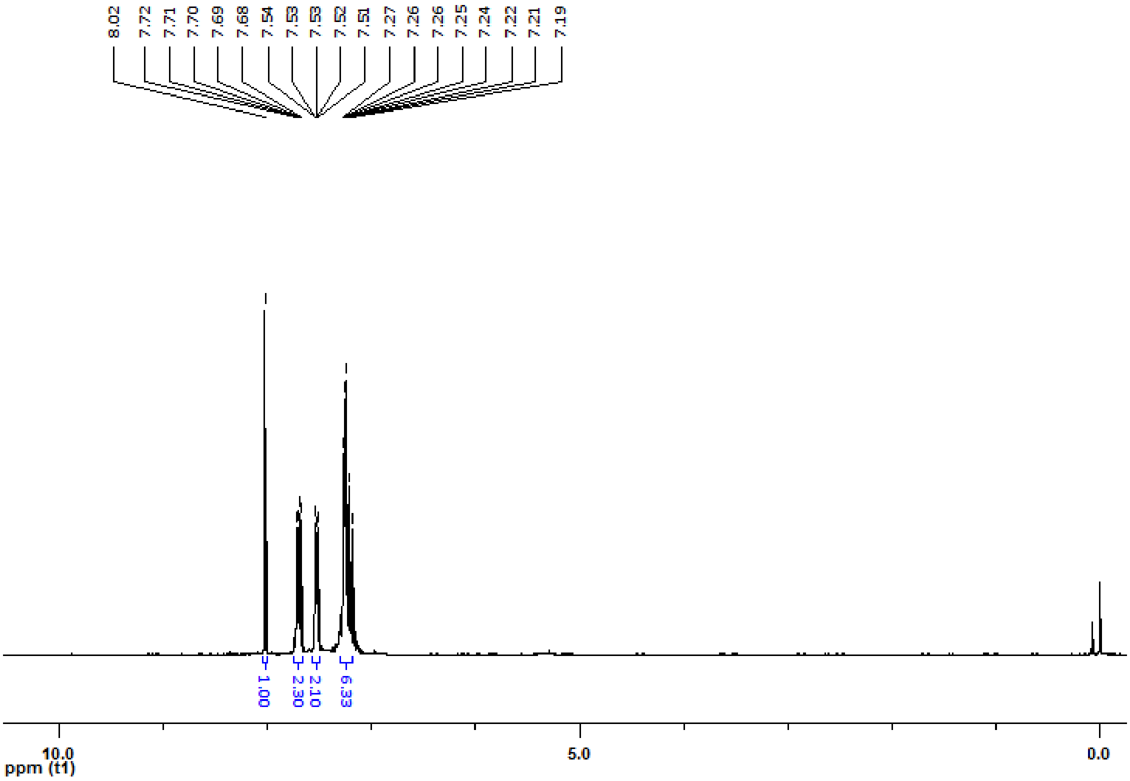

^{1}H NMR (300 MHz, $CDCl_3$) spectrum of **4h**.

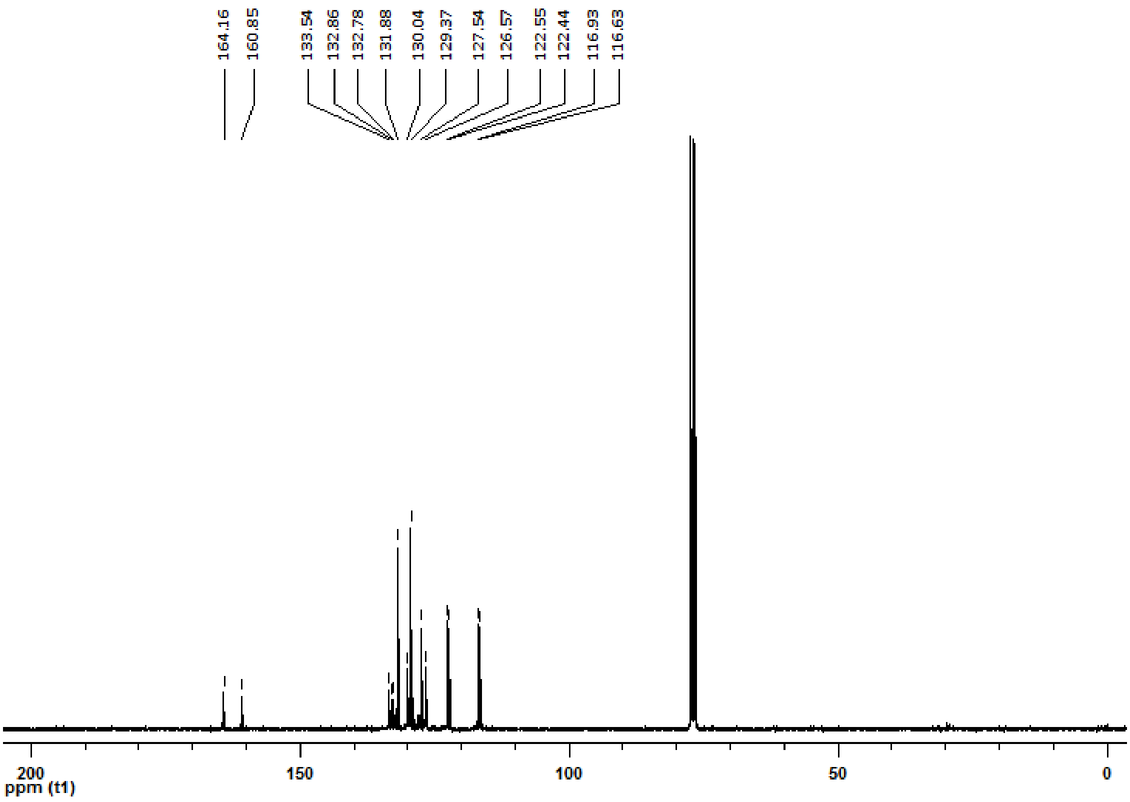

^{13}C NMR (75 MHz, $CDCl_3$) spectrum of **4h**.

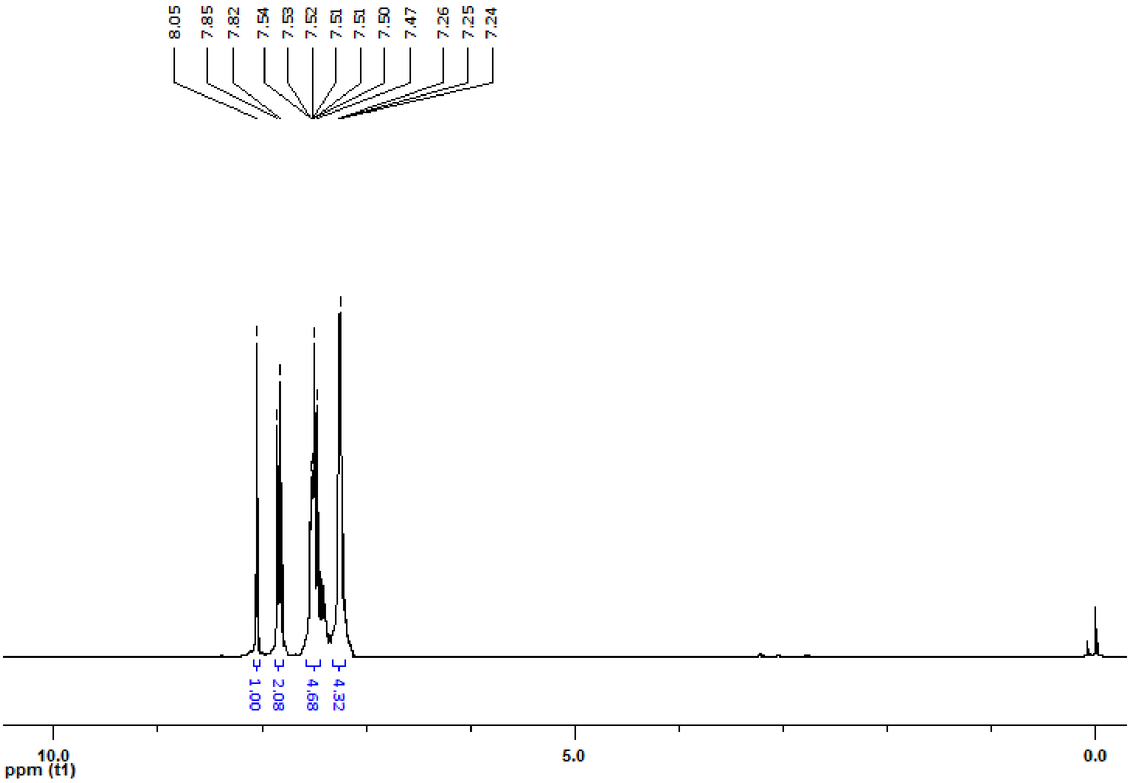

^{1}H NMR (300 MHz, $CDCl_3$) spectrum of **4i**.

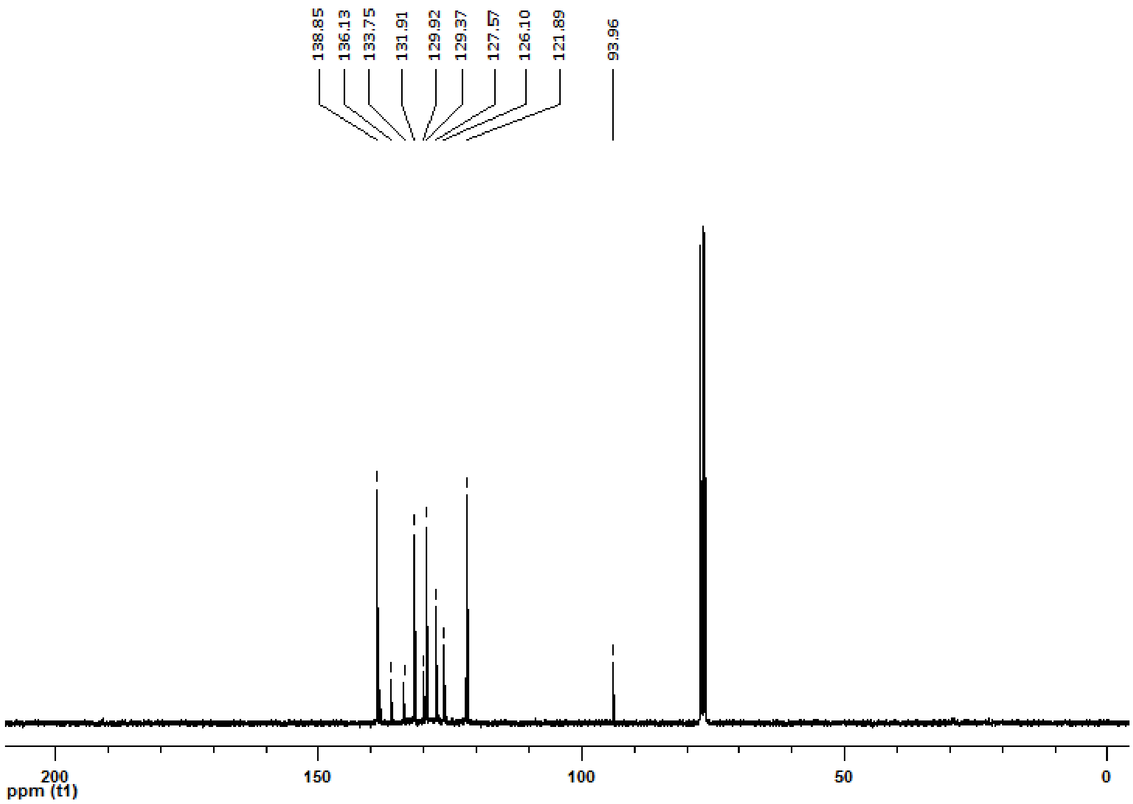

^{13}C NMR (75 MHz, $CDCl_3$) spectrum of **4i**.

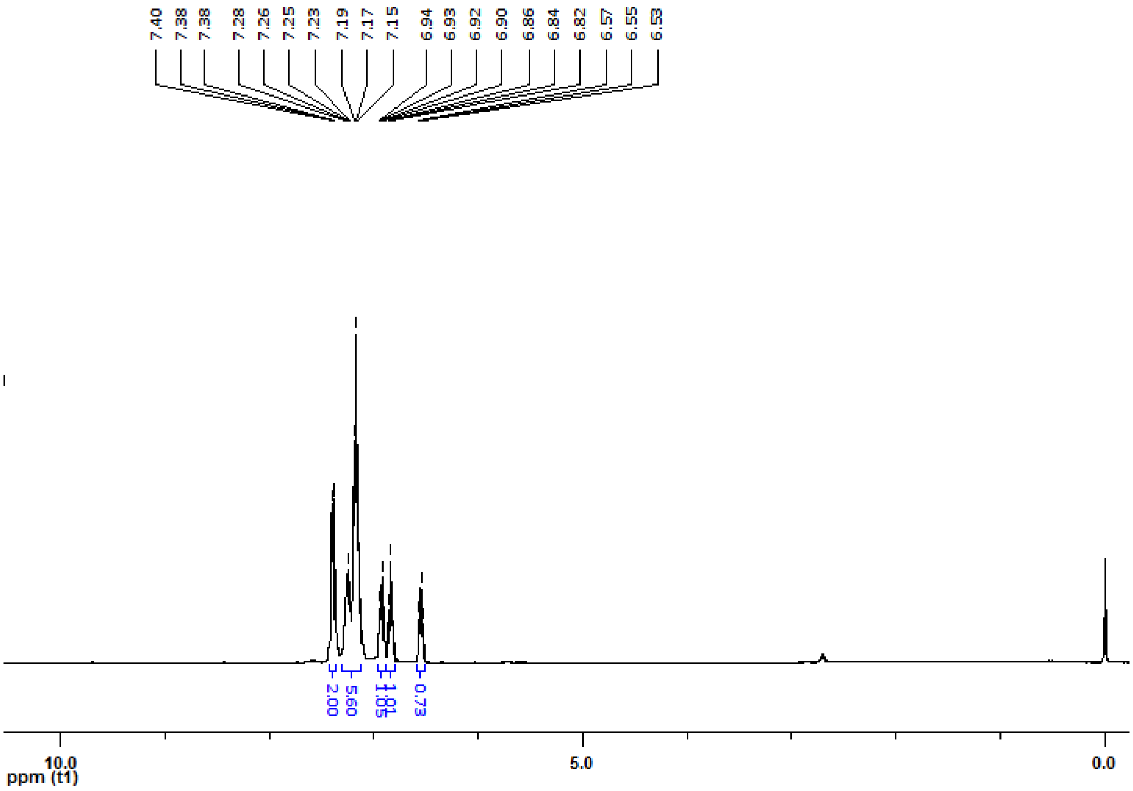

^{1}H NMR (400 MHz, $CDCl_3$) spectrum of **4j**.

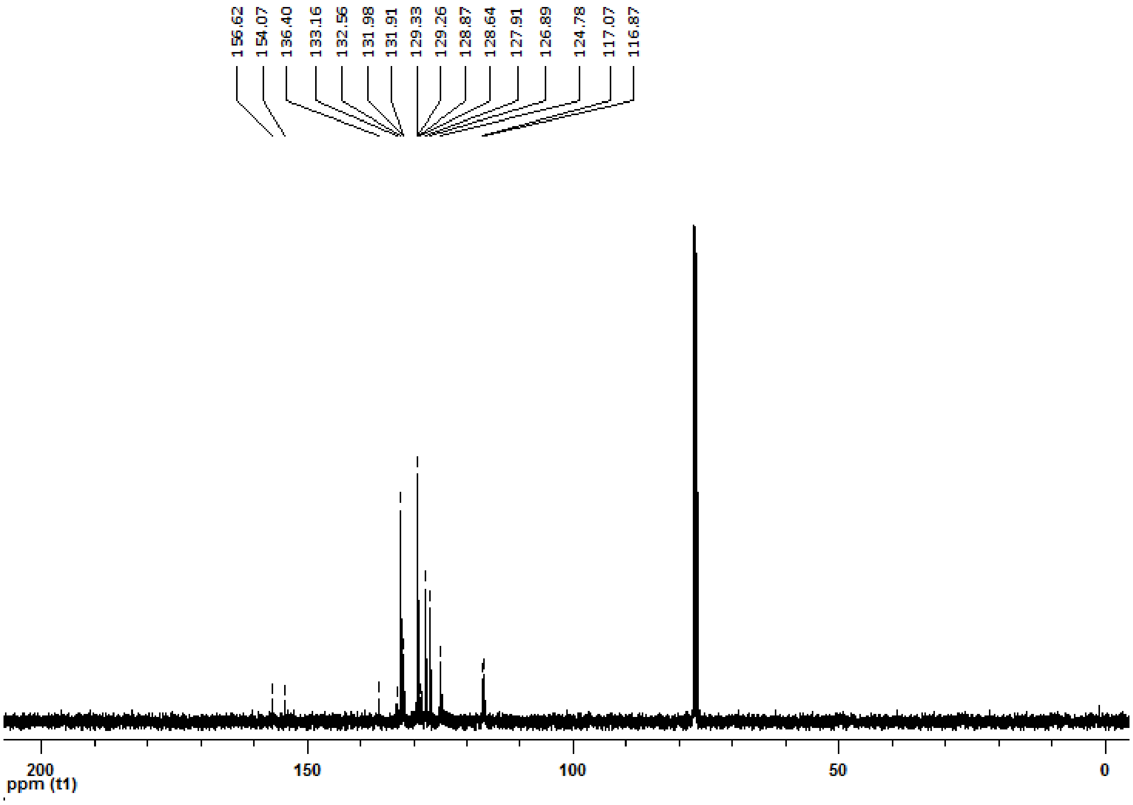

^{13}C NMR (100 MHz, $CDCl_3$) spectrum of **4j**.

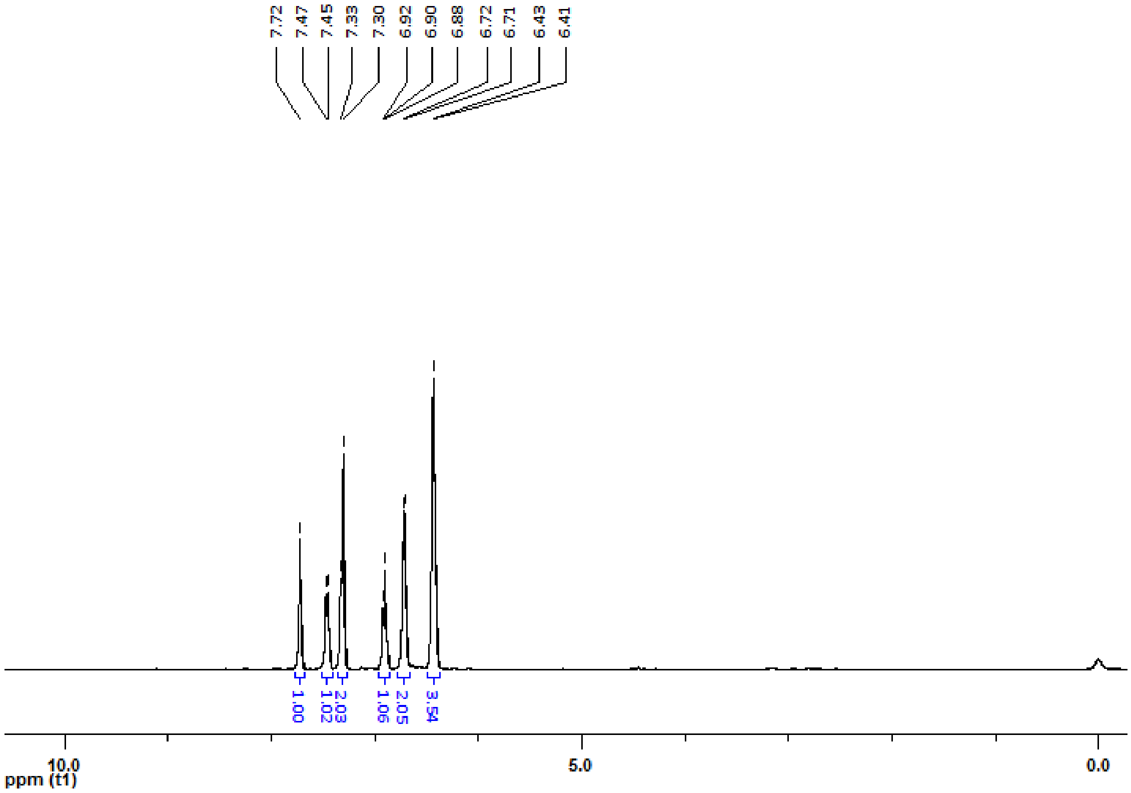

^{1}H NMR (400 MHz, $CDCl_3$) spectrum of **4k**.

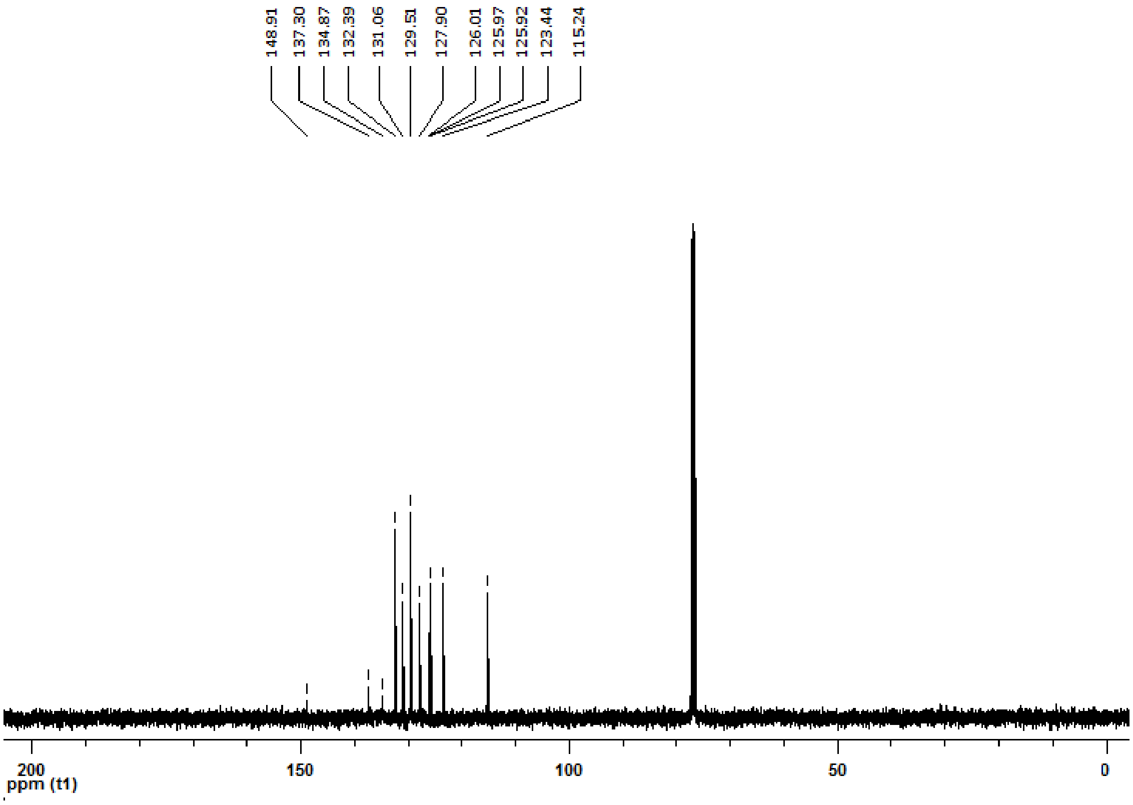

^{13}C NMR (100 MHz, $CDCl_3$) spectrum of **4k**.

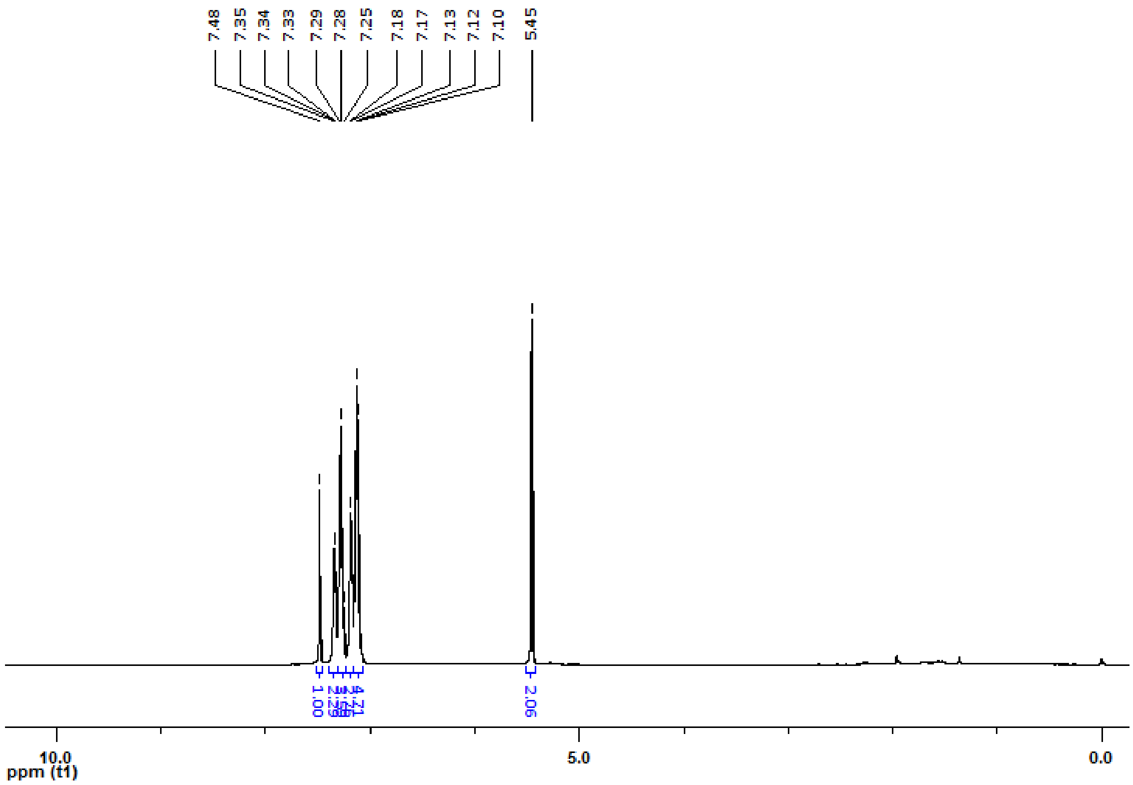

^{1}H NMR (400 MHz, $CDCl_3$) spectrum of **4l**.

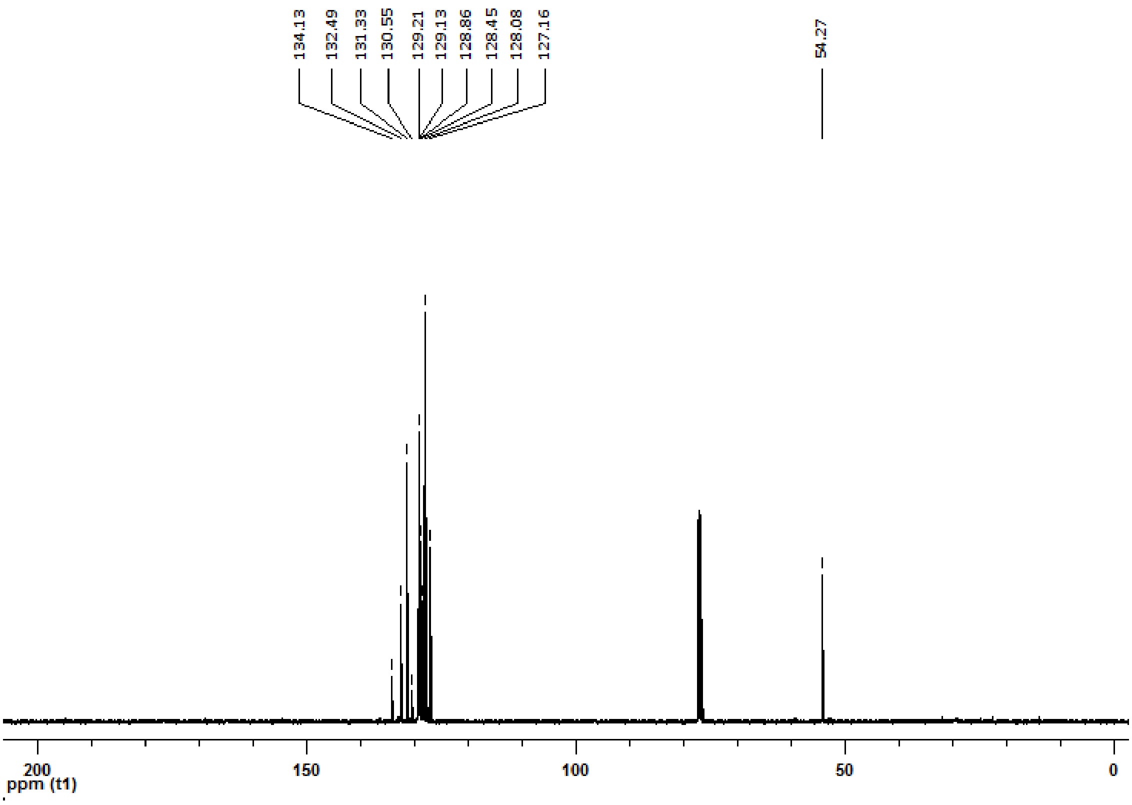

^{13}C NMR (100 MHz, $CDCl_3$) spectrum of **4l**.

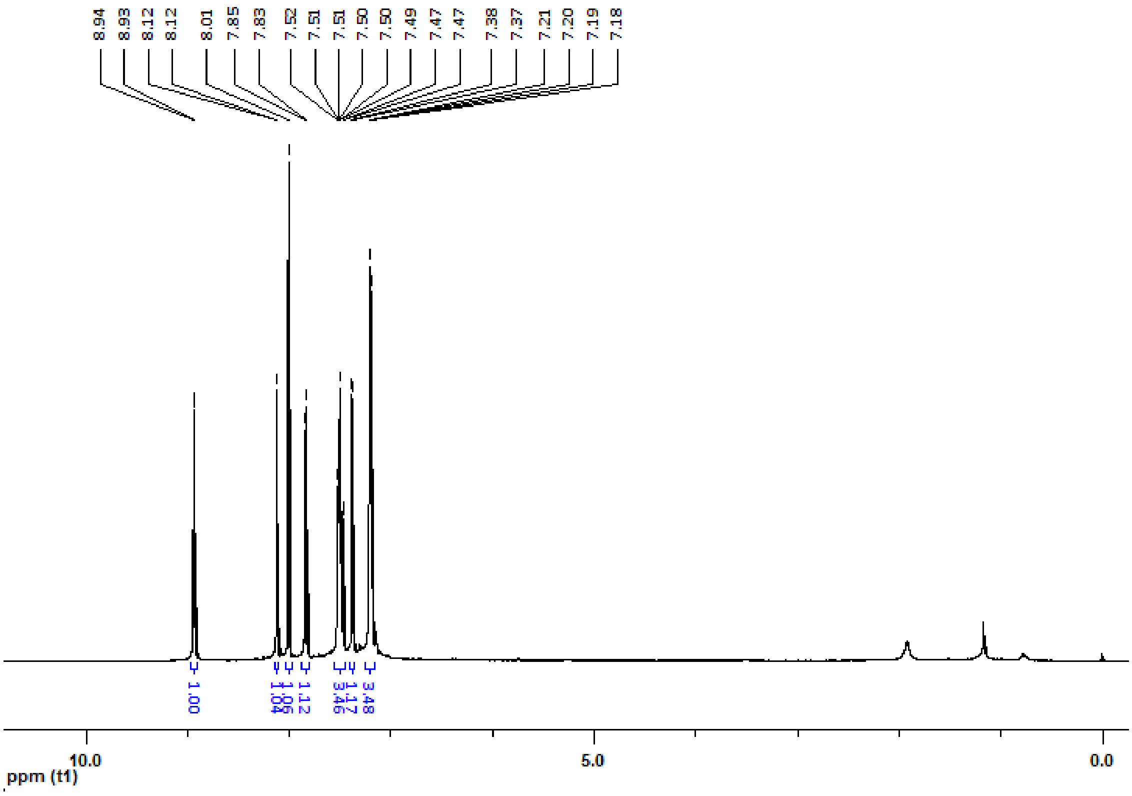

^{1}H NMR (400 MHz, $CDCl_3$) spectrum of **4m**.

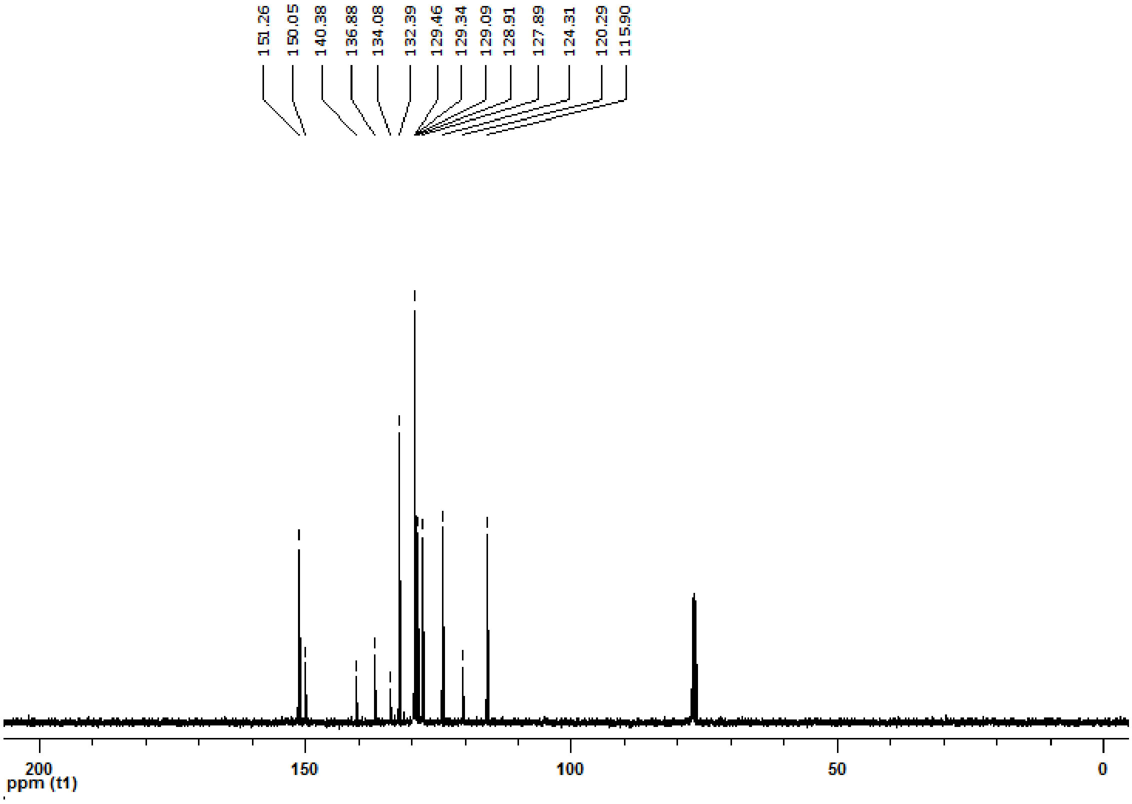

^{13}C NMR (100 MHz, $CDCl_3$) spectrum of **4m**.

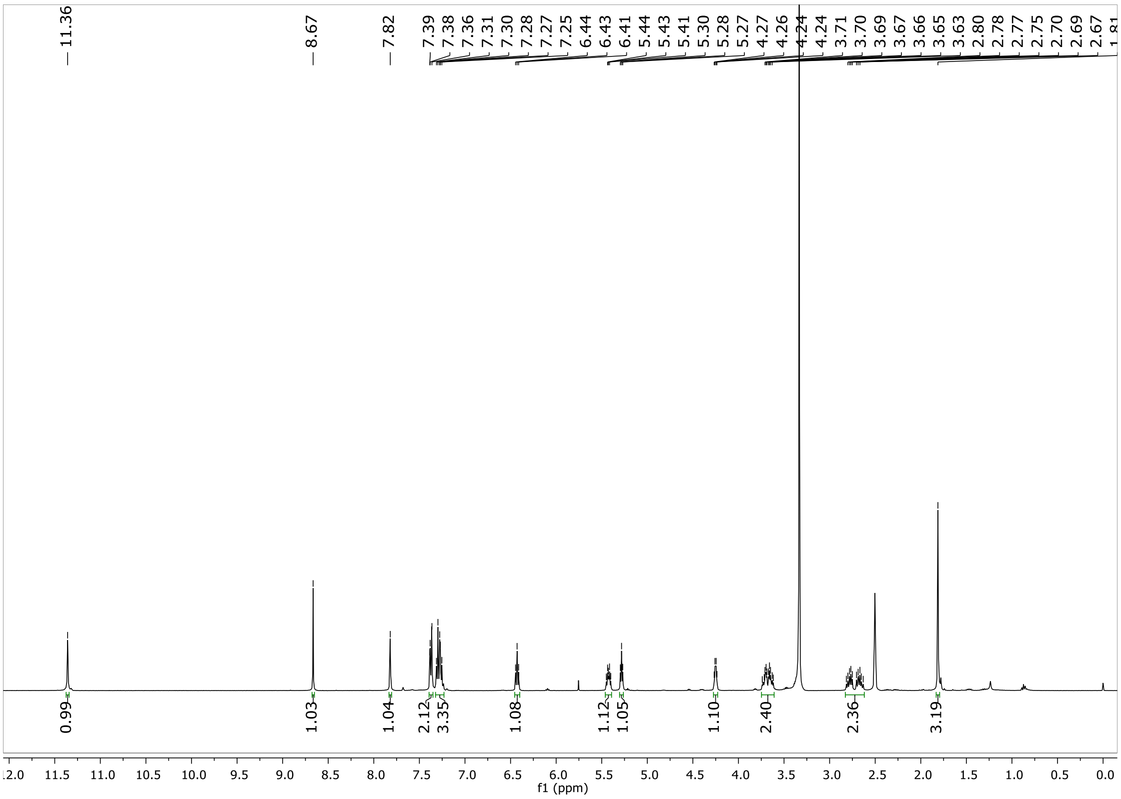

^{1}H NMR (400 MHz, $CDCl_3$) spectrum of **4n**.

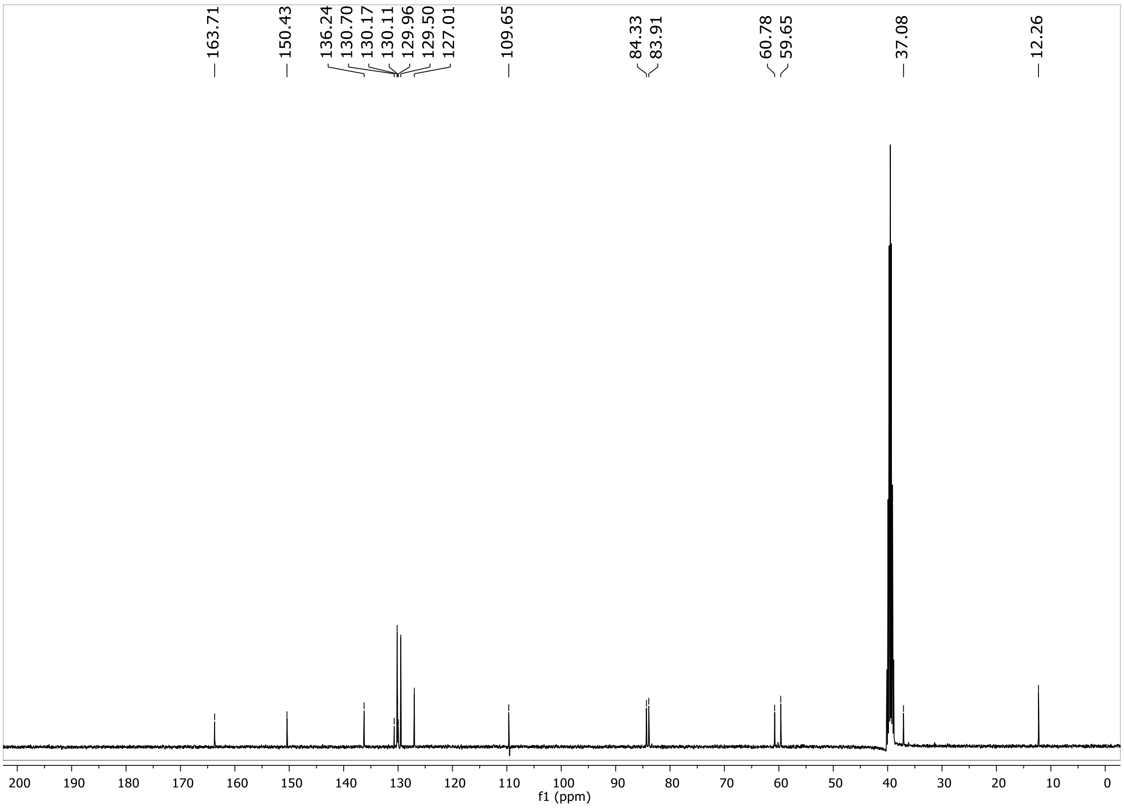

^{13}C NMR (100 MHz, $CDCl_3$) spectrum of **4n**.

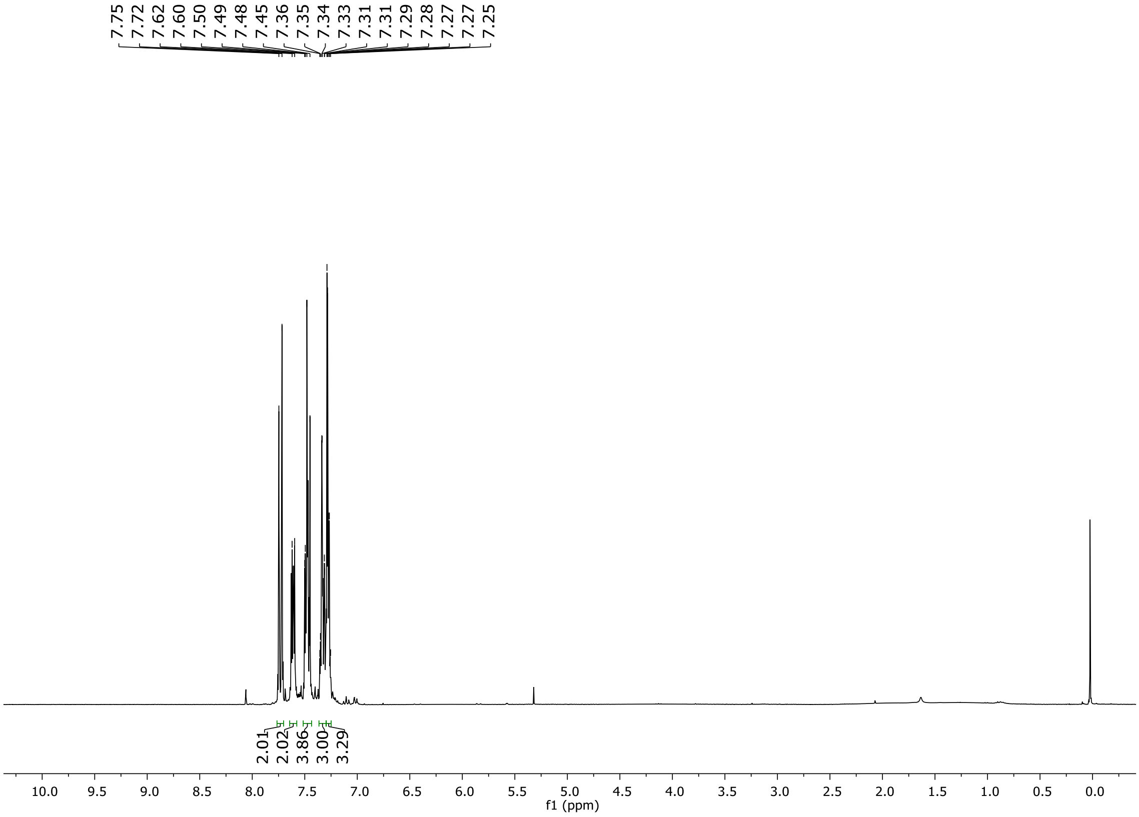

^{1}H NMR (400 MHz, $CDCl_3$) spectrum of **5a**

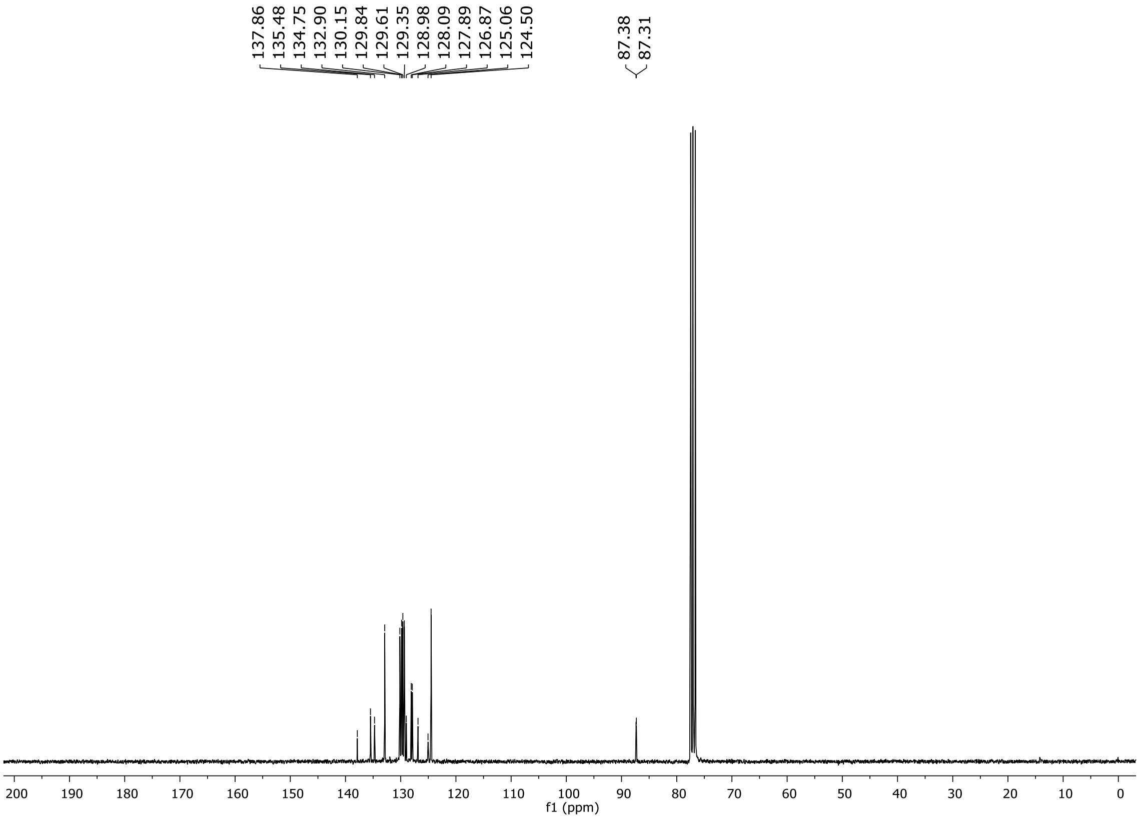

^{13}C NMR (100 MHz, $CDCl_3$) spectrum of **5a**.

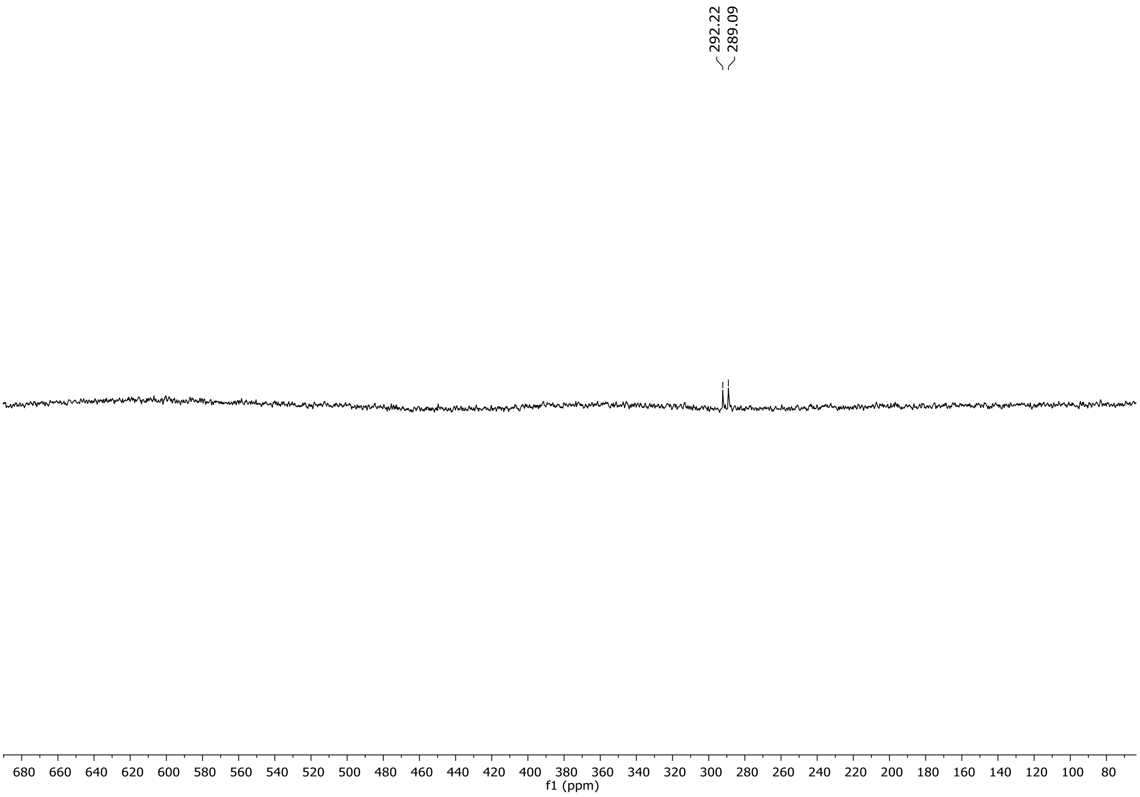

^{77}Se NMR (76 MHz, $CDCl_3$) spectrum of **5a**.

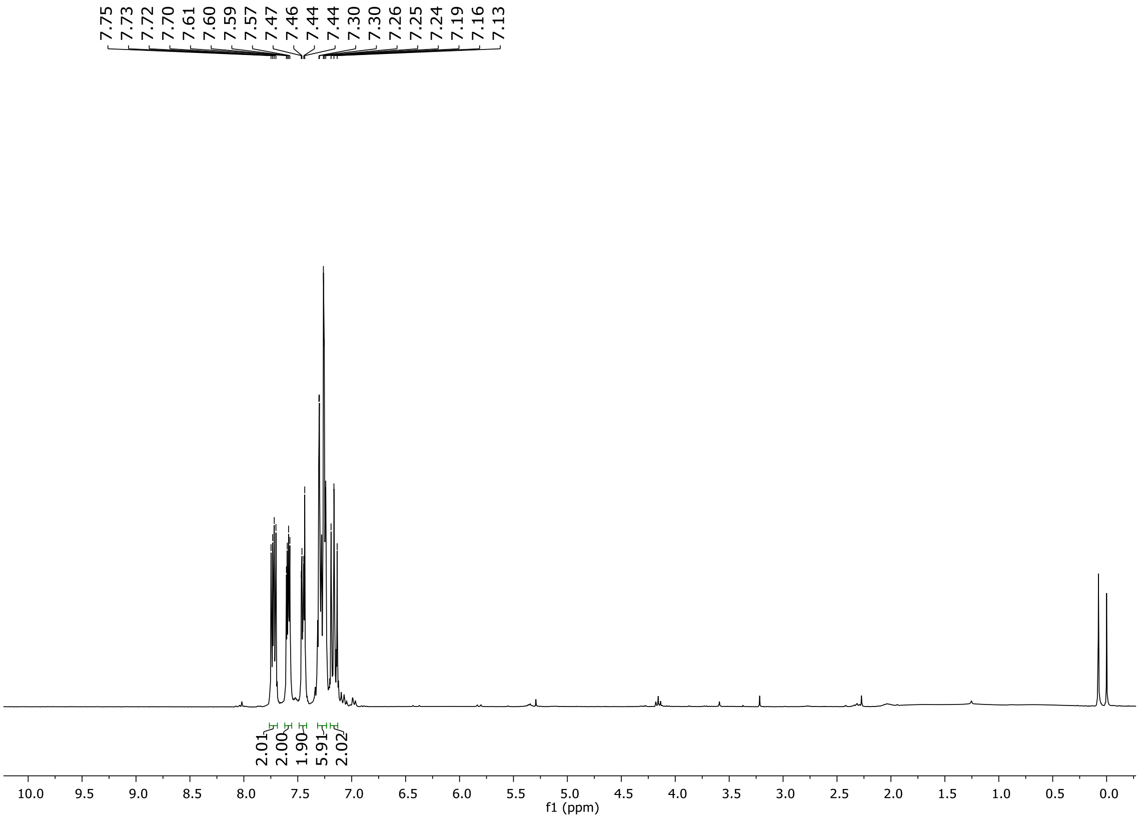

^{1}H NMR (400 MHz, $CDCl_3$) spectrum of **5b**.

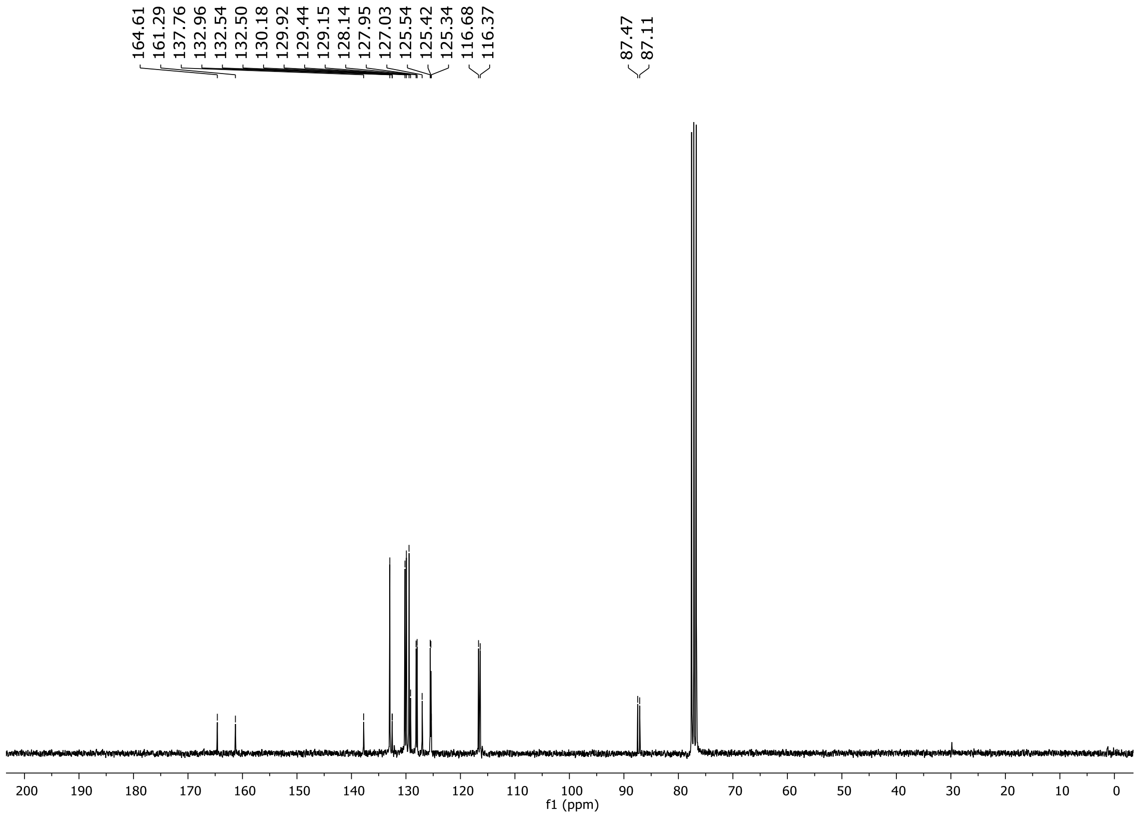

^{13}C NMR (100 MHz, $CDCl_3$) spectrum of **5b**.
